# Crystal structure of the yeast heterodimeric ADAT2/3 deaminase

**DOI:** 10.1186/s12915-020-00920-2

**Published:** 2020-12-03

**Authors:** Xiwen Liu, Ruoyu Chen, Yujie Sun, Ran Chen, Jie Zhou, Qingnan Tian, Xuan Tao, Zhang Zhang, Guan-zheng Luo, Wei Xie

**Affiliations:** 1 MOE Key Laboratory of Gene Function and Regulation, State Key Laboratory for Biocontrol, School of Life Sciences, Sun Yat-sen University, 135 W. Xingang Rd., Guangzhou, 510275 Guangdong People’s Republic of China; 2 Department of Colorectal Surgery, The Sixth Affiliated Hospital, Sun Yat-sen University, 26 Yuancun Erheng Rd., Guangzhou, 510655 Guangdong People’s Republic of China; 3 School of Life Sciences, Zhengzhou University, 100 Kexue Rd., Zhengzhou, 450001 Henan People’s Republic of China; 4 MOE Key Laboratory of Bioinorganic and Synthetic Chemistry, School of Chemistry, Sun Yat-sen University, 135 W. Xingang Rd., Guangzhou, 510275 Guangdong China

**Keywords:** Adenosine-to-inosine editing, tRNA modifications, ADAT, Deaminase, Molecular mechanism

## Abstract

**Background:**

The adenosine-to-inosine (A-to-I) editing in anticodons of tRNAs is critical for wobble base-pairing during translation. This modification is produced via deamination on A34 and catalyzed by the adenosine deaminase acting on tRNA (ADAT) enzyme. Eukaryotic ADATs are heterodimers composed of the catalytic subunit ADAT2 and the structural subunit ADAT3, but their molecular assemblies and catalytic mechanisms are largely unclear.

**Results:**

Here, we report a 2.8-Å crystal structure of *Saccharomyces cerevisiae* ADAT2/3 (ScADAT2/3), revealing its heterodimeric assembly and substrate recognition mechanism. While each subunit clearly contains a domain resembling their prokaryotic homolog TadA, suggesting an evolutionary gene duplication event, they also display accessory domains for additional structural or functional purposes. The N-lobe of ScADAT3 exhibits a positively charged region with a potential role in the recognition and binding of tRNA, supported by our biochemical analysis. Interestingly, ScADAT3 employs its C-terminus to block tRNA’s entry into its pseudo-active site and thus inactivates itself for deamination despite the preservation of a zinc-binding site, a mechanism possibly shared only among yeasts.

**Conclusions:**

Combining the structural with biochemical, bioinformatic, and in vivo functional studies, we propose a stepwise model for the pathway of deamination by ADAT2/3. Our work provides insight into the molecular mechanism of the A-to-I editing by the eukaryotic ADAT heterodimer, especially the role of ADAT3 in catalysis.

## Background

Post-transcriptional modification on RNA is an elegant strategy adopted by nature to diversify gene expression products and expand the functionality of the transcripts. Among various modifications, the adenosine-to-inosine (A-to-I) editing via deamination is a major type that is widely occurring in mRNA and tRNA. In tRNA, this deamination event takes place at positions 34 (the first position of the anticodon) and 37 (immediately 3′ to anticodon). Specifically, I34 allows the decoding of three different nucleotides at the third position of mRNA codons, as inosine is able to pair with U, C, and A. Such expanded base-pairing capability results in an enhancement in the decoding capacity of tRNA and promotes translational efficacy [1]. Therefore, the I34 modification in tRNA is essential for cell viability [2–7].

The enzyme responsible for the conversion of tRNA(A34) is named adenosine deaminase acting on tRNA (ADAT) or tRNA-adenosine deaminase (Tad), and it belongs to the CDA (cytidine deaminase) superfamily. The core catalytic domains of ADATs resemble CDAs and possess the characteristic H(C) XE and PCXXC zinc-binding signature sequences [5]. ADAT enzymes have been characterized from both bacteria and eukarya, but they were found to adopt different structural assemblies. The bacterial enzyme named TadA forms a homodimer. By contrast, in eukaryotic organisms like yeasts, ADAT is a heterodimer formed by ADAT2 and ADAT3 (the ScADAT2/3 heterodimer). ADAT2 is the catalytic subunit, and ADAT3 serves only a structural role, because the essential glutamate in the conserved H(C) XE motif of the latter is usually mutated to a catalytically inactive residue (a valine in the ScADAT3 case) [5]. Correspondingly, tRNA substrate repertoires in the two kingdoms are different. In eukarya, a few tRNAs (seven or eight) with genomically encoded A34 are deaminated by the ADAT2/3 heterodimer [8–11], whereas in bacteria, the A-to-I editing by TadA is found mostly in tRNA^Arg^, although recent studies have shown that other tRNA species could be modified as well [12, 13]. How eukaryotic ADAT2/3s modify various tRNAs remains an open question. Biochemical studies identified that the recognition elements for ScADAT2/3 consisted of the target base A34 and the purine base at position 35 (with the exception of tRNA^Arg(ACG)^), but the structural basis is unclear [9].

Since the determination of the first TadA crystal structure in 2005, i.e., TadA from *Aquifex aeolicus* (AaTadA, PDB 1WWR) [14], TadA structures from multiple bacterial organisms have been solved (PDBs 1WWR, 1Z3A, 2A8N, 2NX8, and 3OCQ) [14–17]. AaTadA displays an α/β/α three-layered fold and forms a homodimer. The dimeric fold is completely different from the tetrameric structure of yeast CDD1 [18], which deaminates mRNA and cytidine, but it resembles the dimeric structure of the yeast cytosine deaminase [14]. The crystal structure of *Staphylococcus aureus* TadA (SaTadA) in complex with tRNA^Arg^ anticodon stem-loop (mini-tRNA) determined by Losey et al. provided further insight into the RNA substrate recognition mechanism of bacterial TadAs (PDB 2B3J) [19]. Because the RNA-binding site of SaTadA overlaps partially with the protein dimerization interface, both protein subunits in the dimer make extensive contacts with the bound RNA molecule bearing a nebularine (an adenosine analog) at position 34. These contacts are made almost exclusively to the five nucleotides of the mini-tRNA loop (U33-G37), with the unusual C32-A38 base-pair capping the ends. Two years later, the structure of the catalytic domain of the human ADAT2 was solved without a publication (PDB 3DH1). But to date, there is no structure available for ADAT heterodimers, which greatly hinders the understanding of the molecular mechanism of A34 deamination in eukaryotes.

In addition to its important role in inosine formation, ADAT3 has been implicated in genetic diseases. The homozygous mutation (c.382 G > A) in human ADAT3, leading to the V144M mutation in the non-catalytic subunit, has been found causative for autosomal-recessive intellectual disability (ID) in 24 affected individuals from eight Arab families [20]. El-Hattab et al. reported another 15 patients carrying the identical homozygous *ADAT3* mutation from 11 Arab families, with similar symptoms [21]. Analysis of tRNA isoacceptors isolated from ID-affected individuals revealed that these tRNAs contained hypomodified I34. Additionally, the cell extract exhibited a dramatic reduction in deaminase activity [22]. However, the underlying structural basis is poorly understood.

In this study, we determined a crystal structure of the ScADAT2/3 heterodimer to 2.8 Å and revealed the assembly of the eukaryotic ADAT enzymes. The structure showed extensive interactions between the two proteins, disruption of which by mutagenesis could greatly reduce deamination activity in vitro and inhibit the function in vivo. Importantly, while the C-terminal half of ScADAT3 forms a pseudo-catalytic domain (PCD), resembling TadA, the eukaryote-specific N-terminal sequence forms a stand-alone domain and functions as a tRNA-binding domain. The relaxed substrate specificity conferred by ScADAT3 partially explains the expansion of tRNA substrates of heterodimeric ADATs. Interestingly, *Saccharomyces cerevisiae* employs a dual mechanism to eliminate the deamination activity from ScADAT3, by both blocking the potential catalytic pocket and replacing the key glutamate residue as the general base. Additional phylogenetic analyses indicated that the same blocking mechanism might be shared only by closely related yeast species, suggesting its ancient origin. Lastly, by combining our structure analyses and biochemical studies, we proposed a model of the deamination pathway for heterodimeric ADAT2/3 enzymes.

## Results

### Overall structure of the ScADAT2/3 heterodimer

**Fig. 1:**
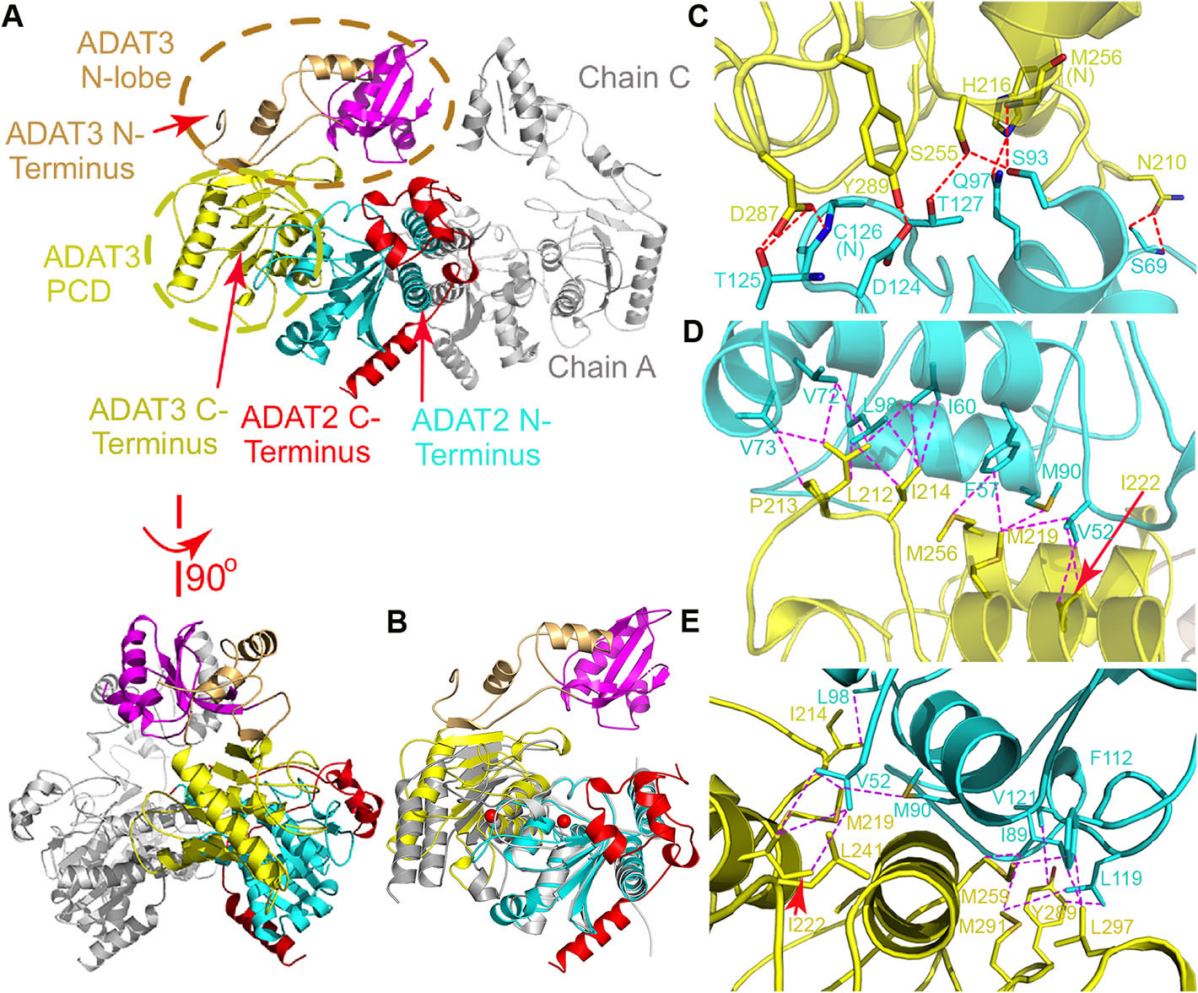
The overall structure of the ScADAT2/3 heterodimer and inter-subunit interactions. **a** The ScADAT2/3 heterodimeric structure shown in the ribbon rendering in two orthogonal views. The N-lobe and PCD of ScADAT3 are colored light orange and yellow, respectively, and circled; RLD is shown in magenta. The catalytic domain (CD) and the C-terminal protrusion of ScADAT2 are colored cyan and red, respectively. The N- and C-termini are indicated. **b** Superimposition of the AaTadA homodimer structure (PDB 1WWR, gray) with that of the ADAT2/3 heterodimer. Their major differences are the additions as the C-terminal tail of ScADAT2 and the N-lobe of ADAT3, which are colored red and magenta, respectively. The zinc ions were shown as the red spheres. **c** The interfacial hydrogen bonding network between the heterodimers as indicated by the red dashed lines (cutoff distance 3.5 Å). **d**, **e** The hydrophobic interactions as indicated by the dashed magenta lines (cutoff distance 4.7 Å)

The co-expressed heterodimer was ~ 90% pure after the cleavage of the N-terminal 6× His-tag. The crystals were grown at 4 °C and would grow to an ideal size over a week. The 4 °C crystallization was essential for crystal growth to avoid protein degradation. The crystals normally diffracted to 3.5 Å, and the best one diffracted to 2.80 Å. The structure was solved by molecular replacement (MR) using the human ADAT2 structure dimer (PDB 3DH1) as the search model. There are two ScADAT2 (chains A and B) and two ScADAT3 copies (chains C and D) in the asymmetric unit, forming two heterodimers. In the final model, the two ScADAT2 monomers are visible from Ala2-Pro165 and Asn178-Val244 for chain A and Met1-Glu162 and Val174-Val244 for chain B, while the two ADAT3 monomers are visible from Pro8-Ile74, Glu84-Cys88, Asp95-Ser105, and Pro122-Cys322 for chain C and Asn7-Ser105 and Lys112-Cys322 for chain D, respectively (Fig. 1a and Additional file 1: Fig. S1). Chain C suffers more disorder than D due to crystal packing. Therefore, we herein focus on the structural information collected from the B/D chain pair because of their more complete structure, unless specified otherwise. Other than the resolved 8097 protein atoms, there are also four zinc ions, one bound by each subunit, as well as 108 water molecules (Additional file 2: Table S1). Structural comparison of the ScADAT2/3 heterodimer to the TadA dimer (PDB 1WWR) revealed that while they could be well superimposed, the former displays extra additions as the C-terminal tail of ScADAT2 and the N-lobe of ScADAT3 (Fig. 1b). ScADAT2 forms a central five-stranded β-sheet, flanked by several helices on each side. The N-terminus of ScADAT2 (Met1-Asn161) forms the catalytic domain (CD), while the C-terminal protrusion (~ 80 residues) forms several peripheral helices connected by long loops. The β4-strand is antiparallel to the other strands, thus forming a mixed sheet. On the other hand, ScADAT3 can be clearly divided into two domains, with the N-lobe (Met1-Asp159) displaying an extended structure. The C-lobe of ScADAT3 (Met160-Cys322) forms PCD (pseudo-catalytic domain), which will be described in the following sections.

### The specific interactions between the heterodimer and their effects on activity

The ScADAT2 and ScADAT3 subunits of the heterodimer make substantial contacts, exclusively through the ScADAT2-CD/ScADAT3-PCD interface, burying a total surface area of 2010 Å^2^. However, the two heterodimers within the same asymmetric unit barely contact each other. All the ScADAT2-CD/ScADAT3-PCD contacts are either hydrogen bonds or hydrophobic interactions, and none of them is mediated through salt bridges. The interfacial hydrogen bonding network involves ten hydrogen bonds. Specifically, the amide group of Asn210 of ScADAT3 accepts a hydrogen bond from the side chain hydroxyl and the backbone amino group of Ser69 from ScADAT2, respectively. Ser255 from ScADAT3 forms two hydrogen bonds with Thr127 and Ser93 from ScADAT2 using its side chains, and the latter subunit also makes a hydrogen bond with the backbone of Met256 from ScADAT3 (Fig. 1c). In addition, the Asp124-Cys126 fragment from ScADAT2 adds four more hydrogen bonds: while the amino group of the ScADAT2/Cys126 backbone and the side chain of ScADAT2/Thr125 donate a total of three hydrogen bonds to ScADAT3/Asp287, ScADAT2/Asp124 accepts one from ScADAT3/Tyr289. Lastly, ScADAT2/Gln97 also accepts one hydrogen bond from ScADAT3/His216. Apart from some sporadic interactions, the hydrophobic interactions are mainly concentrated in two areas of ScADAT3: the Leu212-Ile222 and the Tyr289-Met291 fragments (dashed magenta lines in Fig. 1d, e). The former interacts with Val52, Val72, Val73, Ile60, and Met90 from ADAT2, while the latter forms a network involving Ile89, Phe112, Leu119, and Val121 from ScADAT2 (partial contribution from Met259 and Leu297 of ScADAT3).

**Fig. 2:**
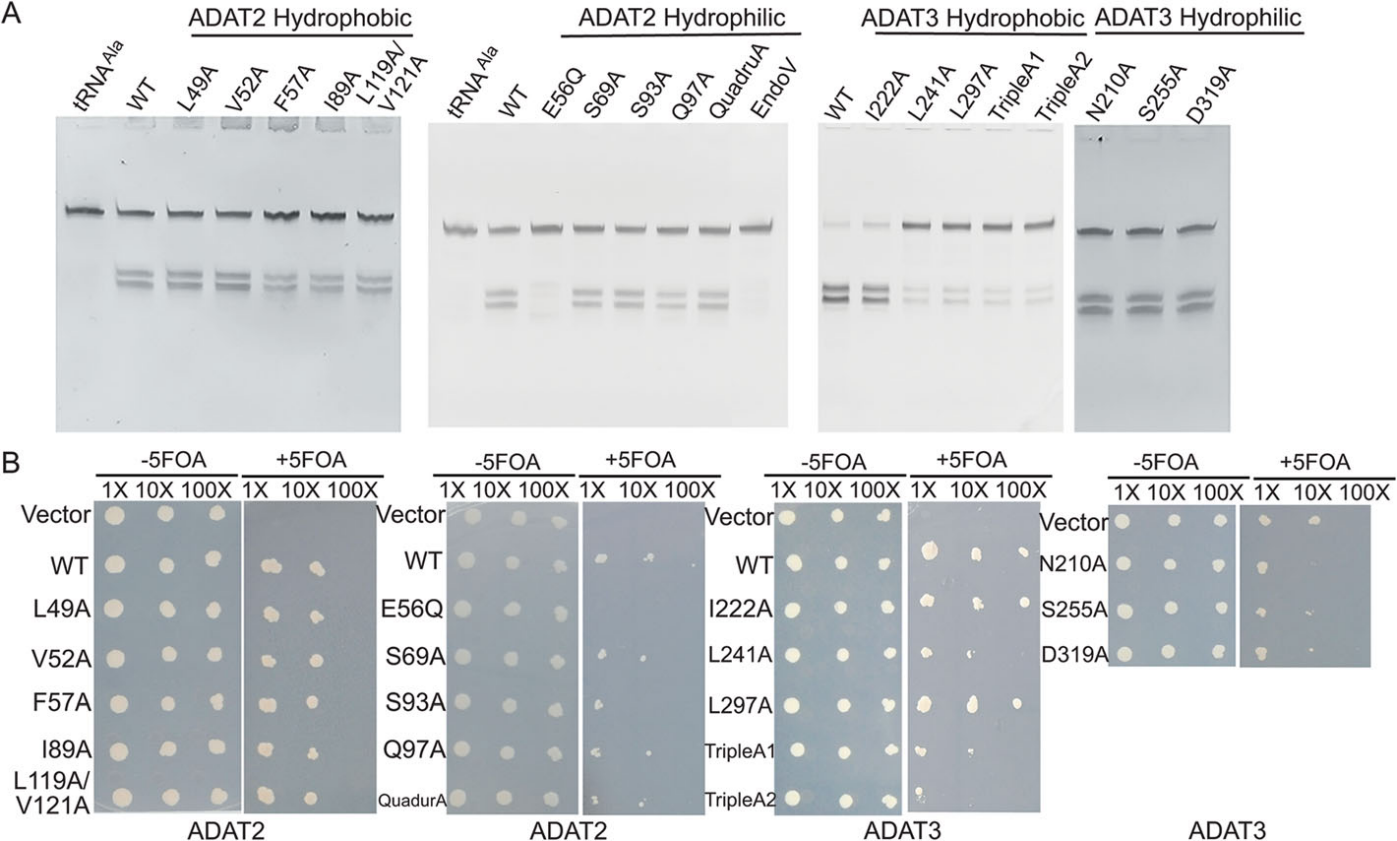
The in vitro and in vivo experiments to test the functions of ScADAT2/3. **a** The in vitro tRNA deamination assay coupled with cleavage by EndoV. The mutants are grouped according to their sources and biophysical properties and are shown above each panel. QuadruA: the ScADAT2-D124A/T125A/C126A/T127A mutant; TripleA1: ScADAT3-L212A/P213A/I214A; TripleA2: ScADAT3-D287A/Y289A/M291A. Note that the cleavage of the tRNA^Ala^ would generate two bands at 35 and 41 nt, respectively. **b** The knocked-out and complementation assays. Each mutant is diluted by 1×, 10×, and 100×, respectively, and 5-FOA is added (+ 5-FOA) or omitted (− 5-FOA) from the media

To examine the structure-activity relationship of the heterodimeric assembly, mutations of ScADAT2 or ScADAT3 concerning these individual interactions were created, and the contribution of the intersubunit contacts to ScADAT2/3’s function was assessed indirectly by enzymatic activity assays. These mutations were created either on ScADAT2 for perturbation of its polar interactions (S69A, S93A, Q97A, D124A/T125A/C126A/T127A (named QuadruA)) or its hydrophobic interactions (L49A, V52A, F57A, I89A, L119A/V121A), or on ScADAT3 for perturbation of its hydrophobic interactions (I222A, L241A, L297A, M259A, L212A/P213A/I214A (TripleA1), D287A/Y289A/M291A (TripleA2)) or its polar interactions (N210A, S255A, D319A). All the mutants were expressed and purified in an identical manner to that of the WT except for M259A, which was not expressed (Additional file 3: Fig. S2). During the assay, the deamination activity of ScADAT2/3 was coupled to that of *Escherichia coli* EndoV (EcEndoV), which recognizes inosine and cleaves the specific phosphodiester bond after position 35 [23]. Pilot experiments revealed that in the presence of 30-nM WT ADAT enzyme and 2-μM EcEndoV, the deaminated tRNA would be completely cleaved by the latter in the specified period (no more cleavage product would be generated if more time is given). In proof of the principle, tRNA^Ala^ with A34 was efficiently cleaved through the tandem reactions by ScADAT2/3 and EcEndoV, while EcEndoV alone generated almost no products (Fig. 2a). In addition, the negative control ScADAT2/E56Q (mutation of the key activating residue) only had residual activity (1.3% of WT). Most mutations on ScADAT2 moderately reduced enzymatic activity, with all of them retaining > 50% activity (Fig. 2a and Additional file 4: Table S2). In contrast, the ScADAT3 mutations involving the hydrophobic interactions produced more dramatic consequences, with four mutations ScADAT3/L241A, ScADAT3/TripleA1, and ScADAT3/TripleA2 severely crippling enzymatic activity. In contrast, mutants of the polar residues barely impacted activities (Fig. 2a).

To further support the results of our in vitro assays, we knocked out the *ADAT2* or *ADAT3* gene separately from *S. cerevisiae* by homologous recombination and complemented the loss of either gene by adding back corresponding mutant-expressing vectors bearing the *ADH1* promoter. Because both the *ADAT2* and *ADAT3* genes are essential to the growth of *S. cerevisiae*, the growth of the knockout strains was maintained by the expression of a helper plasmid harboring a copy of either deleted gene before the complementation, which could be suppressed by the selection of 5-fluoroorotic acid (5-FOA) upon the induction of the mutant genes (Additional file 5: Fig. S3). We found that three mutations on the ScADAT3 hydrophobic residues (L241A, TripleA1, and TripleA2) that crippled in vitro activity also greatly retarded colony growth. Meanwhile, we noted that there were discrepancies between the in vitro deamination tests and the in vivo complementation assays. ScADAT3/L297A, with low in vitro activity, showed normal colony growth, while ScADAT2/S93A, ScADAT3/N210A, and ScADAT3/S255A showed normal in vitro activities but compromised functions in vivo, which reflected the differences in the involved factors between the two distinct evaluation systems. Lastly, to examine the potential mis-folding scenarios caused by the mutations, the mutants were subjected to circular dichroism (CD) analysis (Additional file 6: Fig. S4, and Additional file 7: Table S3). The results showed while the ScADAT3/D319A and ScADAT2/I89A mutations somewhat perturbed the structures, the rest had quite similar fractions of helices and β-sheets as WT, suggesting normal folding patterns.

### The relaxed substrate recognition for ScADAT2/3

**Fig. 3:**
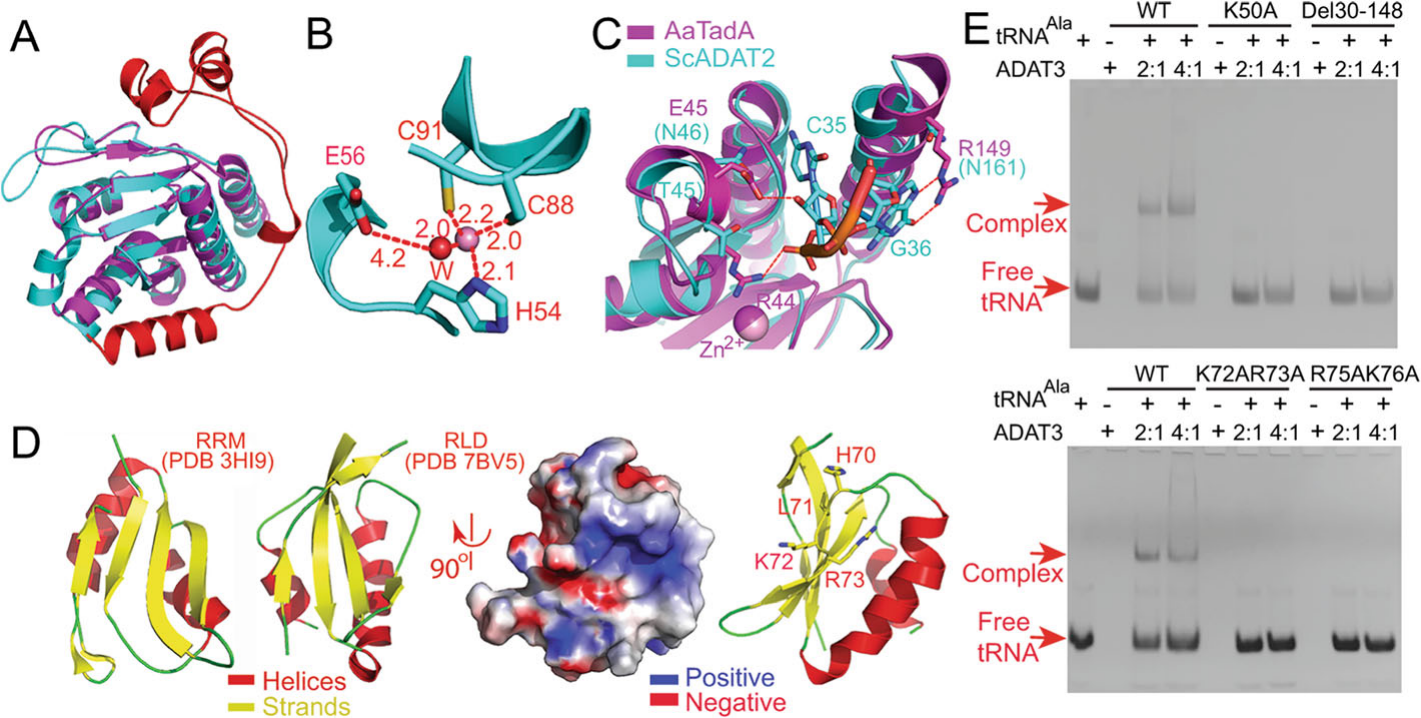
The structural and functional analysis of each subunit. **a** The superimposition of the Cα traces of the ScADAT2 and SaTadA structures (PDB 2B3J, magenta), and the color scheme of ScADAT2 (PDB 7BV5) is as in Fig. 1: catalytic domain in cyan and C-tail in red. **b** The close-up of the zinc-binding site of ScADAT2. The distances are indicated, and the purple and red spheres represent the zinc ion and the catalytic water molecule (W), respectively. **c** The C35-G36 recognition by SaTadA (magenta). The hydrogen bonds are indicated by the red dashed lines. The superimposed ScADAT2 structure is colored cyan. **d** Structural resemblance of a representative of the RRM (left, PDB 3HI9) to that of the RLD (the contents in the box on the right, PDB 7BV5) domains. RLD is shown in both ribbon and surface charge rendering in the same orientation, which are related by a 90° rotation. The 70HLKR73 region is shown in sticks in the cartoon representation (right). **e** The EMSA assay on the binding affinities of the WT and mutant enzymes concerning the tRNA-binding residues in ScADAT3. The molar ratios of the enzyme to tRNA are shown above the gel while bands for the free and enzyme-bound tRNA are indicated on the left

The catalytic domain of ScADAT2 clearly resembles that of SaTadA (PDB 2B3J) [19] (Fig. 3a). A comparison to the SaTadA structure indicates that their RMSD value is 1.4 Å over 147 Cαs. Like SaTadA, the active site of ScADAT2 comprised a zinc ion coordinated by four ligands: His54, Cys88, Cys91, and a water molecule (Fig. 3b). The four ligands form the zinc-binding site, with Glu218 located nearby to activate the water molecule for the nucleophilic attack. SaTadA reportedly catalyzes the sole deamination of tRNA^Arg(AGG)^ [19]. In contrast, ScADAT2/3 acts on seven tRNAs, and our structure offers a partial explanation for this phenomenon. SaTadA makes specific hydrogen bonds with each base in the anticodon loop (C32-A38), and all of them are conserved bases except for G37 [19]. In contrast, except for A34 and purines at position 35, ScADAT2/3 has no other identity determinants [8, 9]. This may be because most key residues in SaTadA for tRNA recognition are replaced in ScADAT2, which would unlikely establish similar specific interactions as SaTadA. Namely, Arg44 and Glu45 in SaTadA are responsible for making contacts with the phosphate and O2’ hydroxyl groups of nucleotide 35, respectively, which are replaced by shorter side-chained threonine and asparagine in ScADAT2. Similarly, Arg149 in SaTadA makes base-specific contacts with G36 and is replaced by Asn161 in ScADAT2 (conserved in eukaryotic ADAT2s), which falls outside the hydrogen bonding distance range (Fig. 3c). Although we could not rule out the possibility of tRNA-induced enzymatic structural changes, the recognition of fewer bases (or even the sole recognition of nucleotides 34–35) by reducing specific contacts with tRNAs would confer broader substrate specificities by ScADAT2/3 than SaTadA.

### The structure and possible function of the N-lobe of ScADAT3

The N-lobe of ScADAT3 (Met1-Asp159) forms a stand-alone domain, suggesting a dedicated purpose. The surface charge rendering of this domain unveiled a patch containing a positively charged region (70HLKR73) that is conserved among the species, which we speculate is possibly used for tRNA binding. Close examination of this region (Lys35-Glu124) reveals that it resembles an RNA recognition motif (RRM), one of the most abundant RNA-binding domains found in eukaryotes (Fig. 3d and Additional file 1: Fig.S1). In addition, this domain (named RLD herein) appears to be generally flexible, as is shown by the higher-than-average disordered regions in this domain in chain C. To examine the tRNA binding ability of RLD, we performed the electrophoresis mobility shift assay (EMSA). Full-length ScADAT2/3 showed a strong binding capacity to the tRNA^Ala^ substrate, but the tRNA binding affinity for the ScADAT3/ΔAsp30-Pro148 mutant (the ScADAT3 N-lobe deletion mutant with the removal of residues Asp30-Pro148 from ScADAT2/3, designated as Del30-148) was completely lost, underscoring the importance of RLD to tRNA binding (Fig. 3e). We also studied the contribution of positively charged regions 70HLKR73 and K50K51 to the binding of tRNA and created the mutations K50A, K72A/R73A, and R75A/K76A separately on ScADAT3. The tRNA binding capabilities for ScADAT3/K72A/R73A and ScADAT3/R75A/K76A were completely lost, as was for the ScADAT3/K50A mutant (Additional file 8: Fig. S5). Additionally, the K50A and Del30-148 mutations also greatly reduced the deamination activities of the enzyme (Additional file 8: Fig. S5). Taken together, we proved that the N-lobe (mainly the RLD domain) contributes significantly to the binding of tRNA substrates.

### The PCD domain of ScADAT3

**Fig. 4:**
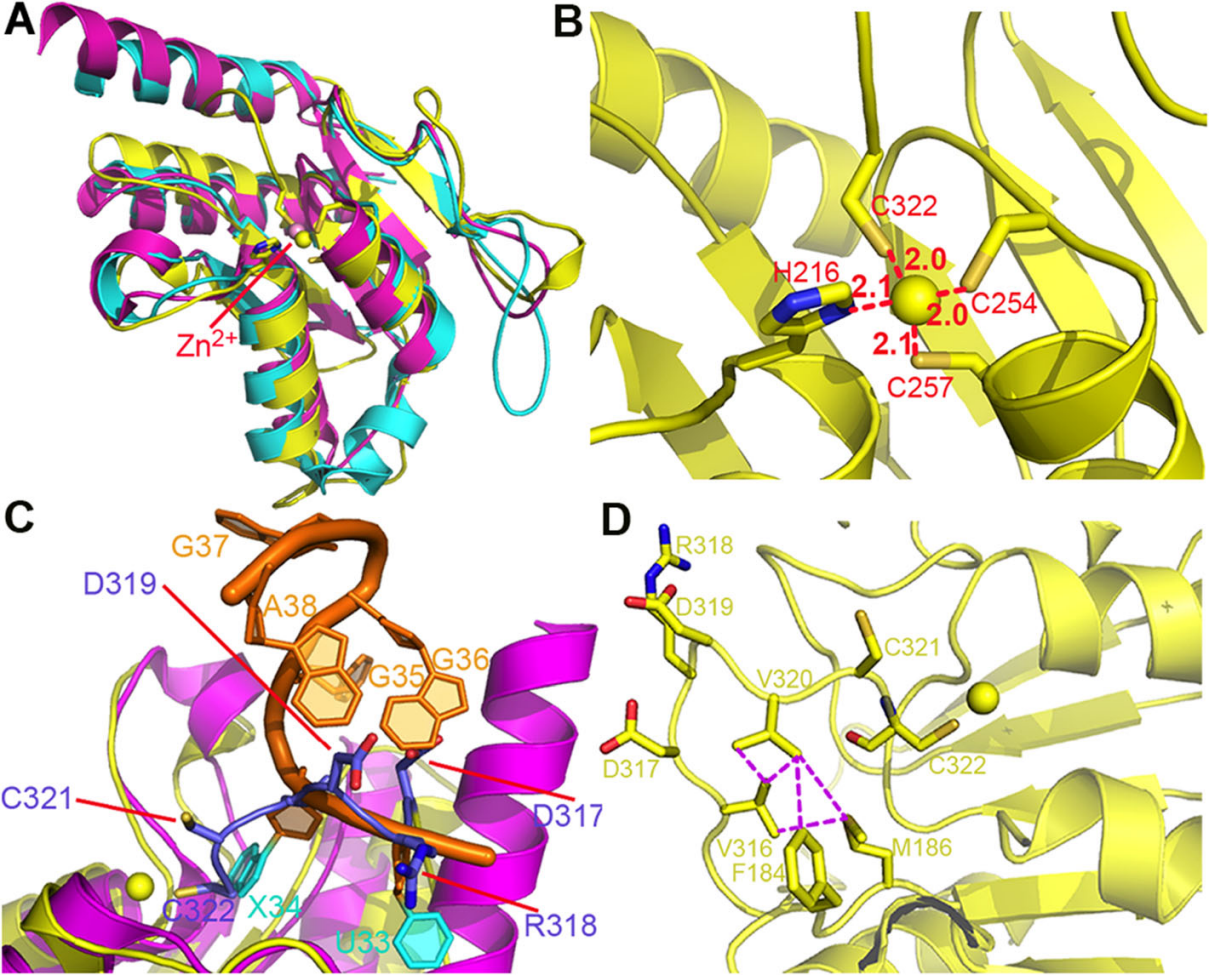
The “blocking” mechanism of ScADAT3. **a** The superimposition of the crystal structure of the yeast ScADAT3 PCD (yellow, PDB 7BV5), ScADAT2 CD (cyan, PDB 7BV5), and SaTadA (PDB 2B3J, magenta). The yellow and pink spheres represent the zinc ions in ScADAT3 PCD and SaTadA, respectively, and the ligands of the former are shown in sticks. Mini-tRNA is omitted for clarity. **b** Close-up of the zinc-binding site of ScADAT3. The distances between the zinc ion to the ligands are shown. **c** The steric clashes between the C-terminal loop of ScADAT3 and the hypothetical tRNA substrate. Coordinates of the RNA substrate (orange) are taken from the SaTadA-RNA complex (PDB 2B3J), and only the C32-A38 fragment is shown. For clarity, the U33-X34 bases of RNA are colored cyan, and the residues Asp317-Cys322 fragment is colored light blue. **d** The hydrophobic interactions formed by the C-terminal loop of ScADAT3. The hydrophobic interactions are shown by the magenta dashed lines

In addition to the structural resemblance between the CD of ScADAT2 and SaTadA, we found that the PCD of ScADAT3 also displays a quite similar fold (Fig. 4a). This domain is so called because the conserved, catalytic glutamate in ScADAT2 catalysis is substituted by a valine (Val218 in ScADAT3), thus abolishing the ability of ScADAT3 to deaminate. In fact, the CD in ScADAT2 and the PCD in ScADAT3 and SaTadA could be well overlaid. We also discovered the zinc-binding site in ScADAT3, which is coordinated by four ligands: His216, Cys254, Cys257, and Cys322 (Fig. 4b). Cys322, the last residue in the ScADAT3 coding sequence, forms the fourth ligand using its side chain and replaces the catalytic water molecule as found in ScADAT2. We tried to restore the activity of ScADAT3 by generating the ScADAT3/V218E mutation, but this heterodimeric mutant was barely soluble in solution. Surprisingly, we found that the tRNA entrance into PCD is blocked by ScADAT3 itself. Specifically, the last fragment of ScADAT3 (Trp306-Cys322) forms a loop and winds back into the pseudo-active site (Fig. 4c). This fragment acts like a “plug” by interacting with surrounding residues mainly through hydrophobic interactions, mediated by ScADAT3/Val316, ScADAT3/Val320, ScADAT3/Phe184, and ScADAT3/Met186 (Fig. 4d). Consequently, it is firmly locked in place, which is supported by relatively lower B-factors of this loop (51.0 Å^2^, comparing to the average of 57.5 Å^2^ for ScADAT3). The positions of residues Arg318-Cys322 in ScADAT3 precisely coincide with bases U33-X34 of the mini-tRNA fragment bound by SaTadA (X stands for nebularine, Fig. 4c) [19]. By sealing the active site, ScADAT3 excludes the binding of tRNA. Furthermore, these two non-conserved cysteines are absolutely important for the stability of the ScADAT2/3, as the Cys321Cys322 deletion from ScADAT3 (ScADAT2/3ΔCC) resulted in the disproportional expression of the two subunits in the *E. coli* host (the intensity ratios of the ScADAT2 to ScADAT3 bands in WT and ΔCC were 0.85 and 0.52, respectively.) (Additional file 9: Fig. S6). Further successive trimming attempts from the C-terminus of the ScADAT3/V218E mutant to make room for tRNA entry were unsuccessful, due to the fact that all truncation mutants were invariably insoluble. Altogether, our structural finding indicated that the C-terminus of ScADAT3 seals its own active site, and the enzymatic structural stability might suffer from the loss of the zinc ligand or the C-terminal hydrophobic loop.

On the other hand, in order to understand the importance of the zinc ion, we also attempted to lose the structural Zn^2+^ ion present in ScADAT3 by introducing three separate mutations to the zinc-binding residues: ScADAT3/C322S, ScADAT3/DelC321-C322, and ScADAT3/C322S/H216A. The first two mutants could be expressed normally, and our inductively coupled plasma mass (ICP-MS) experiments showed that the endogenous zinc ion was still present (data not shown). Our explanation was that a nearby water molecule moves over to fulfill the role of the fourth ligand. However, the C322S/H216A double mutation led to the loss in stability of the enzyme to a substantial extent and resulted in its expression in the insoluble fraction. Therefore, the metal appears to be critical for protein stability or correct folding, and it is not easy to obtain a functional zinc-less ScADAT3. We think that the enzyme so evolves that the terminal cysteine seals the active site and coordinates with the zinc ion simultaneously. This strategy ensures that the C-terminus of ScADAT3 stays in the way of the entering tRNA substrates because the movement of this terminal cysteine (or the C-terminal loop) leads to enzyme instability.

The unexpected terminal cysteine in ScADAT3 acting as the last zinc ligand prompted us the idea to identify possible cysteine candidates for the fourth ligand in other organisms. For example, we carried out the Cys-to-Ser mutations on the cysteine residues of *Schizosaccharomyces pombe* ADAT3 (SpADAT3). Our search criteria are (1) the cysteine would be located in the C-terminal half of the sequence due to the conserved locations of PCD in various ADAT3s and (2) the Cys-to-Ser mutations of the zinc-coordinating cysteines would lead to structural instability of SpADAT3. We therefore mutated five out of the six cysteines located to the C-terminal half of SpADAT3 (315 residues in total without the expression tag), SpADAT3/Cys182, SpADAT3/Cys212, SpADAT3/Cys241, SpADAT3/Cys256, and SpADAT3/Cys271 to serines and tested the expression patterns of these SpADAT3 mutants alone. Cys253 and Cys256 in SpADAT3 are the equivalent residues of Cys254, and Cys257 in ScADAT3, and mutations of SpADAT3/Cys256 as well as SpADAT3/Cys241 resulted in insoluble heterodimeric mutants, presumably all due to the loss of the zinc ligand. On the other hand, the SpADAT3/C182S, SpADAT3/C212S, and SpADAT3/C271S mutants were not remarkably affected in the expression levels and both subunits of the mutants were at least partially soluble. ICP-MS spectrometry measurements revealed that within acceptable errors, all of them still retained the zinc ion (Additional file 10: Table S4). As a control, the WT SpADAT3 contained 1.0 equivalent of zinc ion in molar ratio. Therefore, the most likely candidate for the last zinc ligand would be Cys241 in SpADAT3, which awaits further confirmation by a distinct characterization method. Due to the non-conservancy of the cysteine residue in the ADAT3 protein sequences, lack of structural information of the additional PCD domains, and low throughput of the screen, we terminated our search efforts in other species.

### Phylogenetic analyses

**Fig. 5:**
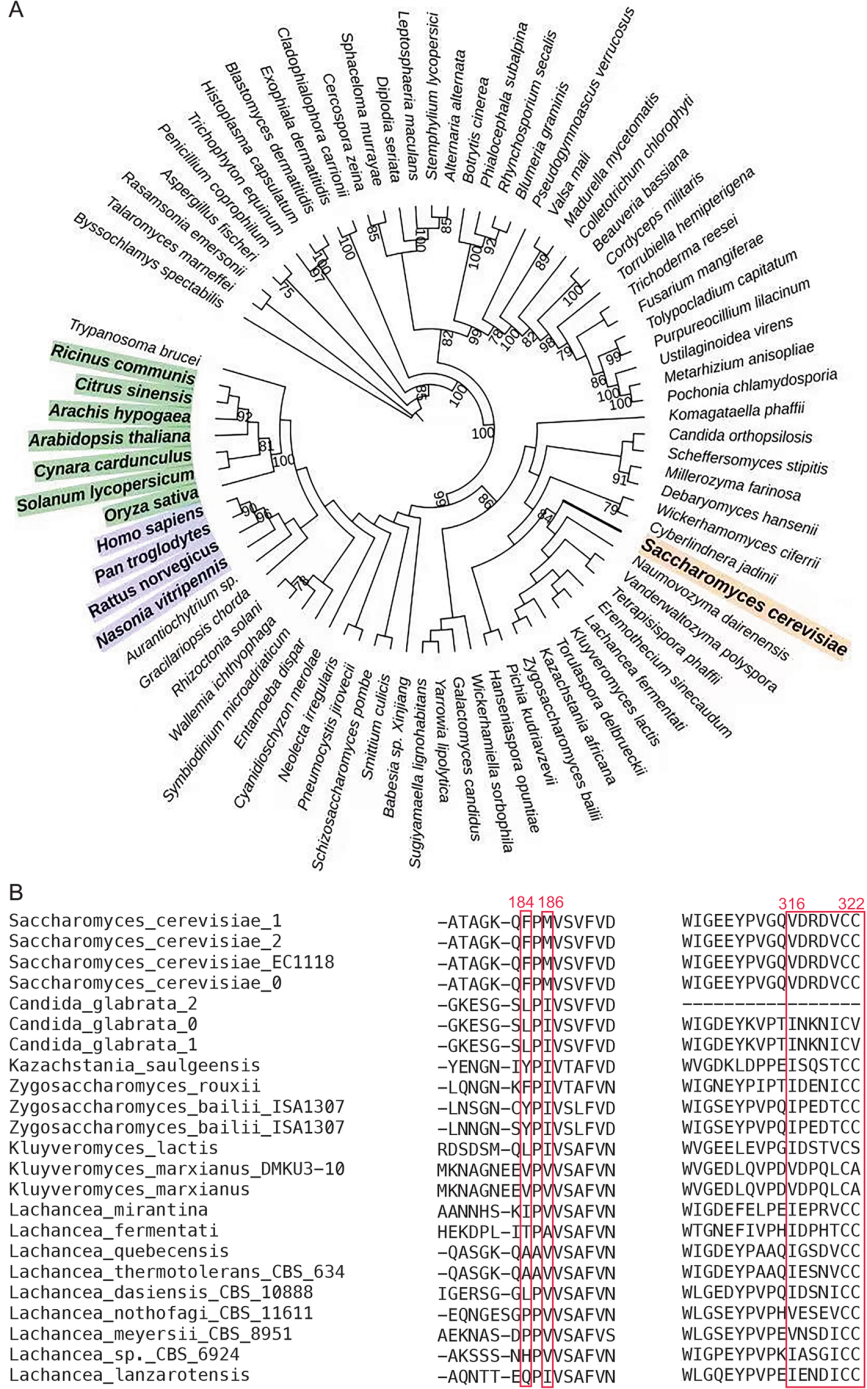
Phylogenetic analyses. **a** The phylogenetic tree of eukaryotic ADAT3s from 151 species. **b** The sequence conservation of the C-termini of various ADAT3s. The residues located in the 184–186 regions (ScADAT3 numbering) and the C-terminal loops for their hydrophobic interactions are boxed

To further examine the conservation of the “blocking” mechanism, we performed phylogenetic analyses on ADAT3. A search for “ADAT3” from NCBI collected 151 sequences in eukaryotes, of which 125 were from Ascomycetes, the largest phylum of fungi. Single-gene phylogenetic analyses were performed on the resulting 81 sequences after removing redundant or partial sequences (Fig. 5a). The two motifs HAX and PCXXC are rather conserved, and the second residues within HAX are normally hydrophilic (Ser/Cys/Ala) while the third residues are usually hydrophobic (Val/Cys) (Additional file 11: Excel Table 1). In contrast to the highly conserved pseudo-catalytic domain, the C-terminal sequences are only conserved within closely related species. Interestingly, an alignment for 23 closely related sequences from yeasts indicated that ADAT3s from several species also end with double cysteines (Fig. 5b). In addition, the two important valine residues for hydrophobic interactions within the last loop (Val316 and Val320) are relatively conserved, or replaced with similar residues like leucines or isoleucines, while their interacting residues Phe184 and Met186 are semi-conserved and hydrophobic in nature as well (possible residues in the corresponding positions are Phe/Leu/Tyr and Ile/Val, respectively). Therefore, the structural hindrance is likely shared by these closely related yeasts, where the last loop fixed by the hydrophobic interactions within PCD prevents the binding of tRNA while the last cysteine acts as the fourth ligand.

### Implications in disease

**Fig. 6.**
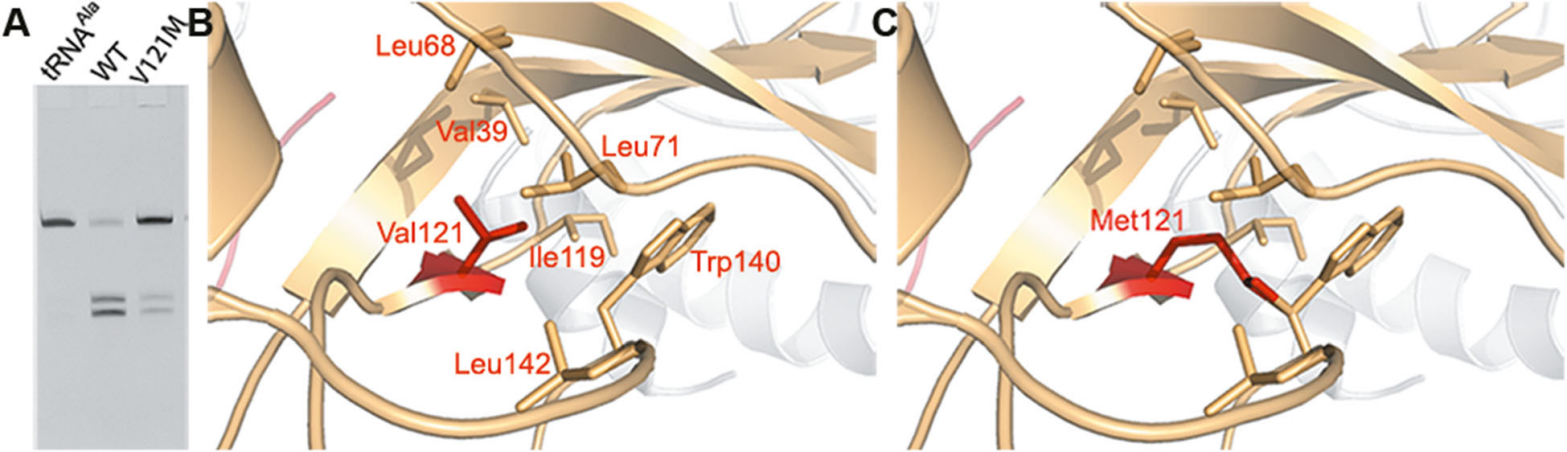
The tRNA deamination activity test of ScADAT3/V121M and the structural basis for disease. **a** The deamination assays on WT and ScADAT3/V121M. **b** The surrounding environment of ScADAT3/Val121. The residues that may establish hydrophobic contacts with Val121 are shown and labeled. **c** The potential steric clashes in the modeled ScADAT3/V121M mutant

The ADAT3/V144M mutation in humans has been found causative for autosomal-recessive intellectual disability. The valine in position 144 of human ADAT3 is conserved in the *S. cerevisiae* counterpart (ScADAT3/Val121). We therefore introduced this Val-to-Met mutation into ScADAT3. The mutation caused a large decrease in the expression level of the heterodimeric enzyme. Our deamination analysis indicated that the mutant only retained 12.4% activity of that of WT (Fig. 6a and Additional file 4: Table S2), and this large loss in activity was consistent with the results reported by Ramos et al. [22]. Our follow-up structural analysis demonstrated that this residue is located on a β-strand of the N-lobe (β7) near the heterodimer interface and is surrounded by quite a few hydrophobic residues such as ScADAT3/Val39, ScADAT3/Leu68, ScADAT3/Leu71, ScADAT3/Ile119, ScADAT3/Trp140, and ScADAT3/Leu142 (Fig. 6b), all of which form close van der Waals contacts with ScADAT3/Val121 (all within ~ 4.5 Å distance). However, the ScADAT3/V121M mutation would lead to direct steric clashes with the bulky residue ScADAT3/Trp140 (Fig. 6c), which would affect the local stability of this region. In humans, the Trp140 residue is conserved (Trp147 in human ADAT3), while the equivalent residues of Leu71 and Ile119 are leucine and valine in humans, respectively. Considering the fact that the mutation causes the propensity of the human enzyme to aggregate, a similar mechanism is likely to apply to the human ADAT3/V144M mutation.

## Discussion

With the capacity to pair with three different bases (A/U/C), the wobble site inosine partially accounts for the degeneracy of genetic code. Eukaryotic species have been reported to prefer base-pairing with tRNA(I34) [24]. In the present work, we solved the structure of ScADAT2/3, the first heterodimeric ADAT structure from eukaryotes. ScADAT2 is quite a hydrophobic protein, explaining its propensity to precipitate in our expression attempts of numerous recombinant constructs. Secondary structure predication annotated ~ 10% of the ScADAT3 sequence as unstructured regions, suggesting its general flexibility. ScADAT2 could only be co-expressed with ScADAT3, which enhanced the solubility of the former, an observation supported by our structural analysis on the heterodimer interactions. Meanwhile, ScADAT3 alone is prone to degradation, and it also needs the stabilization and protection effects from ScADAT2. To push the resolution limit of the ScADAT2/3 crystals during the structural determination, we cut off the resolution where CC_*1/2*_ for the highest resolution shell was 0.57 (*I*/*σ*_(*I*)_ ~ 1.4). In the finished structure, the structure for two ScADAT2 and one ScADAT3 subunits are nearly resolved completely, but ~ 10% region in the other ScADAT3 subunit (chain C) is disordered (all within RLD).

Following the structural determination, we carried out both in vitro and in vivo assays to characterize the contribution from the inter-subunit interactions of the heterodimeric assembly, but the results from the two assays were not completely consistent somehow. The in vitro deamination assay showed a strong dependence on the hydrophobic residues of ScADAT3 mediating the interactions with ScADAT2, which suggests their critical roles in maintaining the structural stability of the complex. On the other hand, we observed inconsistencies for some of the mutants between their performances in vitro and in vivo. Here, we think that the protein stabilities may play a role in these discrepancies: mutants that have stability problems may be subjected to the treatments of both the in vivo degradation and rescue mechanisms. For instance, mutants remained active in vitro may become susceptible to the cellular degradation machinery while poorly stable mutants in vitro may be salvaged by the chaperone systems in vivo. The actual mechanism awaits further investigation. Work by Auxilien et al. suggested that only one deaminase was responsible for the formation of inosine 34 in all A34-containing tRNAs in yeasts, but the study was inconclusive due to the usage of the partially purified enzyme and only limited selection of unmodified tRNA substrate [8–11].

Upon structural comparison, we found that the residues in TadA responsible for the recognition of anticodon loop bases are mostly mutated in ScADAT2. We propose that this evolutionary divergence of ScADAT2/3 from the homodimeric TadA potentially allowed the expansion of its substrate repertoire. Dissection of the ScADAT3 functions allowed us to identify its N-lobe as a tRNA-binding domain. Apart from the extension domains, the PCD structure of ScADAT3 is quite intriguing, which evolves a “blocking” mechanism to fix the C-terminal loop.

We next tried to identify cysteine candidates as the fourth ligands in organisms whose C-terminal cysteines are missing. The terminal cysteines are only shared among closely related fungi species, suggesting that this is a feature of ancient ADTA3s. The replacement of the catalytic water with the last residue of ScADAT3 is a smart strategy to inactivate this subunit, as the removal or mutation of the cysteine causes instability of the deaminase. However, the Glu-to-Val mutation at the active site is a more stable and reliable means to eliminate the activity of the enzyme. Once established, ADAT3s could abandon the substrate-exclusion mechanism over evolution, thus explaining the absence of the C-terminal cysteines in higher eukaryotes.

**Fig. 7:**
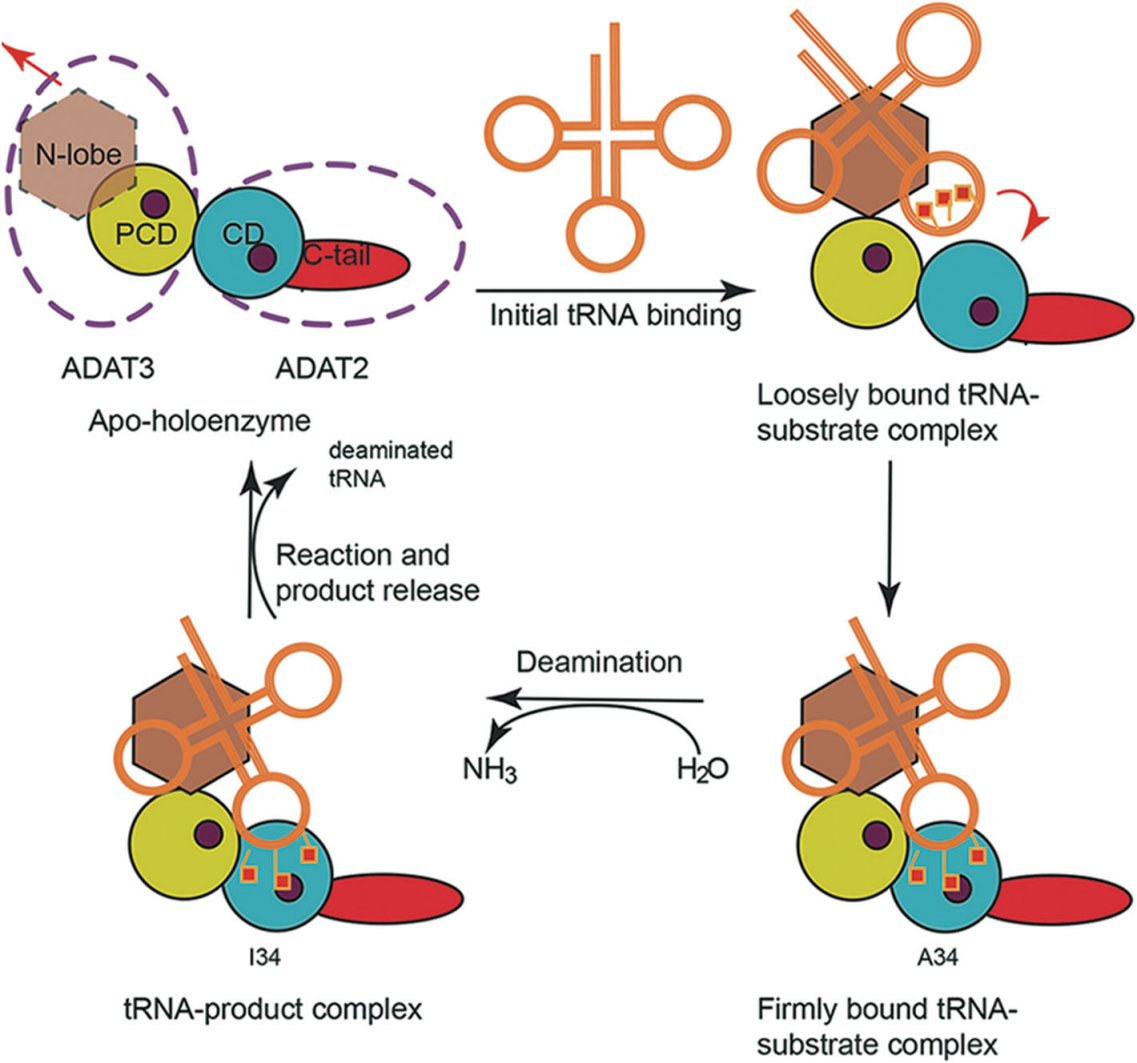
The deamination model. The spheres represent zinc-binding sites in the heterodimeric enzyme. The arrows indicate the movement of the domains during the reaction

Based on our structural and biochemical characterization, we proposed the following reaction pathway by ScADAT2/3 (Fig. 7). (1) The generally flexible N-lobe of ScADAT3 binds tRNA first, followed by the binding of the anticodon loop region to the ScADAT2 subunit. In this step, both the heterodimeric enzyme and tRNA molecules may need to make structural reorganization to form more specific contacts, such as flipping the target base A34 along other bases into the active site. A34 forms an extensive network of hydrogen bonding and van der Waals interactions with ScADAT2, including hydrogen bonds to the conserved residue ScADAT2/Ala55 and Asn43, which are conserved interactions observed in the SaTadA-RNA complex (PDB 2B3J). The adjacent nucleoside at position 35 also makes contacts to assist tRNA positioning. (2) ScADAT2/Glu56 activates the water ligand by abstracting its proton, which directs the nucleophilic attack of the OH^−^ moiety on C6 of A34. Ammonia is then released, and inosine is subsequently formed. (3) The enzyme turns the product over and regenerates itself. We are currently unclear about the stoichiometry of tRNA/ScADAT2/3, and only one tRNA is drawn in Fig. 7, although TbADAT2/3 has been shown to form two types of complexes with its cognate tRNA substrates [25]. Direct support of the model warrants the structures of the enzyme bound with its tRNA substrate at different catalytic stages.

The catalytic property exhibited by ADAT2/3 is reminiscent of many two-subunit enzyme systems such as Trm9/Trm112, Trm11/Trm112, Trm8/Trm82, and Trm7/Trm732. Many of these enzymes are methyltransferases for tRNA modifications. For example, the Trm6/Trm61 methyltransferase is responsible for the methylation at N1 of A58 in tRNAs. This modification is essential to *S. cerevisiae*, due to its enhanced stability of the initiator tRNA. The heterodimer comprises catalytic subunit Trm61 and the non-catalytic subunit Trm6. Trm6 shares homology with Trm61 but is devoid of the conserved SAM-binding pocket. The Trm6-Trm61-tRNA ternary complex structure revealed that the tRNA molecule binds across the interface of each heterodimer. A58 is inserted into the active site of Trm61, orientated by Trm6 from the opposing heterodimer [26]. Therefore, Trm6 works in trans in the heterodimer and is repurposed as a tRNA-binding subunit. A more recent example of heterodimeric enzymes is the human METTL3/METTL14 methyltransferase, the *N*^6^-methyladenosine (m^6^A) writer. Both proteins barely exhibit methyltransferase activity alone, but together, the heterodimer displays strong methyltransferase activity [27].

The involvement of ADAT3 in neurodevelopmental disorders suggests under-estimated significance of this accessory subunit to the functions of the deaminase. The mutation causes structural stability, which was consistent with our serial truncation experiment on ScADAT3 and the mutagenesis trials at the dimer interface. Consequently, while the ScADAT2/3 heterodimer utilizes the ScADAT2 subunit for catalysis through the “half active-site” mechanism, the ScADAT3 subunit contributes to the substrate-binding function and also the stability of the deaminase heterodimer.

## Conclusions

We revealed the substrate recognition and inhibition mechanism adopted by the ScADAT2/3 deaminase. To our knowledge, the self-inhibition phenomenon of a tRNA deaminase is unprecedented. The general conception on ScADAT3 is that it is a non-catalytic subunit, and our mechanistic studies provide the molecular mechanism of its inactivation. Our structure revealed the tRNA-binding role of the N-lobe and the support of ScADAT3 to the catalytic subunit, which has been overlooked or paid little attention in the past. In addition, our studies shed light on the catalytic pathway and the evolutionary origin of eukaryotic ADAT2/3s.

## Methods

### Cloning, protein expression, and purification

The *ADAT2* and *ADAT3* genes (gene IDs 853417 and 851027) were amplified by PCR from *S. cerevisiae* cDNA using the primers ADAT3-F1/R1 and ADAT2-F1/R1, respectively. When the double digestion by the corresponding restriction enzymes was completed, the two genes were sequentially ligated into the multiple cloning site (MCS) of the pETDuet-1 vector (Merck). After *ADAT3* was inserted into MCS1 using the *Eco*RI and *Hind*III restriction sites, *ADAT2* was inserted into MCS2 of the same vector using the *BgI*II and *Xho*I sites. Then, the entire DNA fragment carrying both genes was further amplified from the vector using the primers ADAT3-F2 and ADAT2-R1 and subcloned into the modified pET-28a (+) vector (Merck), in which the thrombin protease cleavage site was replaced by the PreScission protease cleavage site preceding the His6-tag (designated as pET-28a/ADAT2-3). The mutations were created through *Quikchange* PCRs based on the pET-28a/ADAT2-3 vector. All the primers used in this study were listed in Additional file 12: Table S5.

The expression plasmids of pET-28a/ADAT2/3 or mutants were transformed into *E. coli* strain BL21 (DE3) cells for overexpression of the target proteins. The transformed cells were cultured overnight in Luria-Bertani broth containing 50 mg L^−1^ kanamycin at 37 °C. A 4-l fresh culture medium was inoculated with 50 ml of the overnight culture. When OD_600_ reached 0.6–0.8 at 37 °C, the expression of the target proteins was induced by 0.2 mM isopropyl β-d-thiogalactopyranoside (IPTG), and the culture was kept shaking overnight at 22 °C. The cells were then pelleted by centrifugation at 3000*g* for 20 min and resuspended in pre-chilled nickel-nitrilotriacetic acid (Ni-NTA) buffer A containing 40 mM Tris-HCl (pH 8.0), 250 mM NaCl, 10 mM imidazole, 1 mM β-mercaptoethanol, and 1 mM phenylmethylsulfonyl fluoride (PMSF). The resuspended cells were lysed by ultrasonication, and the supernatant was obtained by centrifugation at 23,500*g* for 1 h at 4 °C. The supernatant was then applied onto Ni-NTA affinity resin (Qiagen) pre-equilibrated with Ni-NTA buffer A. The target protein was eluted with Ni-NTA buffer B containing 40 mM Tris-HCl (pH 8.0), 250 mM NaCl, 250 mM imidazole, 1 mM β-mercaptoethanol, and 1 mM PMSF. The 6× His-tag at the N-terminus was cleaved off by being treated with protease overnight at 4 °C in the presence of 5 mM β-mercaptoethanol, which was subsequently applied onto a Histrap column (GE Healthcare) to remove uncut protein. The unbound portion was pooled and dialyzed against a buffer containing 20 mM Tris-HCl (pH 8.0), 250 mM NaCl, and 1 mM DTT. The protein was further concentrated to 2.5 mg ml^−1^, flash cooled in liquid nitrogen, and stored at − 80 °C. For proteins used in activity assays, the WT and mutant proteins were supplemented with 10% glycerol before storage.

### In vitro transcription of tRNA

The full-length tRNAs were prepared by in vitro transcription [28]. The RNA pellet was redissolved in the TE buffer (10 mM Tris-HCl (pH 8.0) and 1 mM EDTA) to a concentration of 5 mg ml^−1^ and annealed.

### Crystallization, data collection, and structure determination

The screens for cocrystals were set up at 4 °C using the sitting-drop vapor diffusion method with the protein concentration of 2.5 mg/ml. Protein crystals were obtained after 1 week under the condition of 20–25% PEG 400 and 0.1 M MES (pH 6.0–6.5). Fully grown crystals were flash-frozen in liquid nitrogen after being soaked in a cryoprotectant containing the reservoir solution supplemented with 20% glycerol (v/v). Diffraction data were collected using Beamline 19U (BL19U1) at the Shanghai Synchrotron Radiation Facility (SSRF, Shanghai, People’s Republic of China) [29] and were processed with the program HKL2000 [30]. The crystals belong to the *P*2_1_2_1_2 space group with 2.80-Å resolution, and the asymmetric unit was predicted to contain two heterodimers. Molecular replacement (MR) was first performed with *Phenix* [31] using the human ADAT2 structure (PDB 3DH1) as the search model. After a plausible solution was obtained, the model was manually built by *COOT* [32] according to the electron density map. The rebuilt model was fed to the *phenix.refine* [31], and multiple cycles of refinement were conducted, followed by model rebuilding. The *R*_free_/*R*_work_ factors were 0.254 and 0.201, and the final model was validated by *Molprobity* [33]. The structural representation of ScADAT2/3 (PDB 7BV5) was prepared by *PyMOL* (http://www.pymol.org/).

### Coupled deamination assay

Two-micromolar tRNA was incubated with 30 nM enzyme in a reaction buffer of 10 mM Tris-HCl (pH 8.0), 25 mM KCl, 2.5 mM MgCl_2_, and 1 mM DTT at 30 °C for 30 min. To this mixture, 0.5-μl CHES (1 M, pH 9.5) and 2 μM EcEndoV [34] were added, and the reaction was incubated at 37 °C for 45 min. The reaction was stopped by 2× urea loading buffer, and the product was analyzed by 15% urea-PAGE gel electrophoresis followed by staining with ethidium bromide and UV light exposure. The software Image J2X was used for data analysis. The cleavage bands generated by EcEndoV were considered as the background, which was deducted correspondingly from the signal generated by each mutant.

### *ADAT2/3* gene knockout and complementation assay

A functional copy of the *ADAT2* or *ADAT3* gene was each first cloned into the helper plasmid pRS426 containing the ADH1 promoter and the marker gene *URA3* (encoding orotidine-5′-phosphate decarboxylase) and was transfected into the *S. cerevisiae* BY4741 strain. Strains expressing this enzyme convert 5-fluoroorotic acid (5-FOA) into toxic 5-fluorouracil and would not survive. PCR-amplified fragments bearing the *Met15* marker gene flanked by upstream and downstream homologous arms from *ADAT2* and *ADAT3* gene sequences (partial intron was used in the *ADAT3* case) and the termination sequence were transfected, and subsequently integrated into the BY4741 chromosome through homologous recombination. Methionine prototrophs were selected and correct genomic integration of the *Met15* gene was verified by PCR and DNA sequencing. Next, the pGADT7 plasmids (with a *Leu* marker gene) carrying the WT or mutant *ADAT2* or *ADAT3* genes were transfected into BY4741. The cells containing the three aforementioned marker genes were selected on plates with the synthetic dropout (SD/-Ura-Leu-Met) medium. The resulting monoclones were grown in 5 ml of liquid culture containing the same medium for 3 days at 30 °C. The absorbance value at 600 nm for each cell suspension was adjusted to 1.0, which was then diluted 10×, 100×, respectively. 0.3 μl of the culture with different dilutions was spotted on the synthetic dropout medium (SD/-Leu-Met+Ura+5-FOA) plates and allowed to grow at 37 °C for further observation.

### EMSA

Two-micromolar tRNA was incubated with ScADAT2/3 at different molar ratios in a buffer of 20 mM Tris-HCl (pH 8.0) and 1 mM DTT on ice for 60 min. The reaction mixture was then mixed with glycerol (10% v/v) before being loaded and analyzed by 6% native PAGE gel with a running buffer containing 40 mM Tris-HCl (pH 9.0). Two gels were run in parallel and electrophoresed at 100 V for 60 min. The gels were stained either with ethidium bromide or Coomassie brilliant blue as designated.

### ICP-MS

Sixty microliters (200 μg) of protein was first treated with 35% HNO_3_ at 65 °C for 2 h and followed by dilution to 5 ml with 1% HNO_3_. The stoichiometry of the zinc ion was determined using an iCAP Q ICP-MS spectrometer (Thermo Scientific) following the instructions. Standard curves were generated using 1% HNO_3_ serial dilutions of known zinc standards. Each measurement represented three technical replicates.

### CD analysis

The WT and mutant proteins were first diluted to a concentration of 0.3 mg ml^−1^ in the buffer containing 20 mM Tris-H_2_SO_4_, 250 mM Na_2_SO_4_, and 1 mM DTT. Far-UV CD measurements were performed on a J-810 spectropolarimeter (Jasco, Japan) using a Teflon-sealed quartz glass cuvette (1-mm path length, Starna, UK). A wavelength increment of 1 nm, a response time of 4 s, and a scan speed of 20 nm/min were used during the measurements. All the resulting spectra were buffer-corrected, and the data were analyzed by the software CDPro [35].

### Phylogenetic analyses

A search was made from NCBI with the key phrase “tRNA-specific adenosine deaminase subunit TAD3,” and 417 proteins in eukaryotes were collected. Similar sequences, sequences too short (< 200 residues) or too long (> 600 residues) were discarded. One sequence from a genus was picked as a representative and 81 sequences remained. The protein sequences were aligned using the Muscle software (v3.8.1551) [36]. The phylogenetic tree was constructed using the maximum likelihood method and WAG model in RAxML (v8.2.12) [37] with 100 bootstraps. The phylogenetic tree was revised by the online software iTOL (http://itol.embl.de/) [38].

## Supplementary Information

**Additional file 1: Fig. S1** The secondary structures and multiple sequence alignments of the two subunits of ScADAT2/3.

**Additional file 2: Table S1** Data collection and refinement statistics.

**Additional file 3: Fig. S2** Purified WT ScADAT2/3 complexes and mutants used for crystallography, activity assays and EMSA.

**Additional file 4: Table S2** The quantification of the deamination activity assays.

**Additional file 5: Fig. S3** schematic diagram showing the knockout/complementation protocols.

**Additional file 6: Fig. S4** The circular dichroism spectra of WT ScADAT2/3 and mutants.

**Additional file 7: Table S3** The circular dichroism analysis on the ScADAT2/3 mutants.

**Additional file 8: Fig. S5** The in-vitro tRNA deamination assay on potential tRNA-binding residues within RLD.

**Additional file 9: Fig. S6** The co-expression/co-purification profile of the ScADAT2/3 WT and ScADAT2/3ΔCC truncation mutant.

**Additional file 10: Table S4** Expression and ICP-MS profile of the Cys-to-Ser mutants of SpADAT3.

**Additional file 11: **Excel **Table 1** The HAX and PCXXC motifs in different species.

**Additional file 12: Table S5** Sequences of the primers and tRNA used in this study.

## Data Availability

All data on which the conclusions are based are present either in the manuscript itself, its additional files, or the Protein Data Bank. The atomic coordinates and structure factors have been deposited in the Protein Data Bank with the accession code 7BV5.
