# Supplementary material for: Crystal structure of the yeast heterodimeric ADAT2/3 deaminase
Source: BMC Biol. 2020 Dec 3;18:189. doi: 10.1186/s12915-020-00920-2 (PMC7713142; doi:10.1186/s12915-020-00920-2)
Supplement: Supplementary file 1 — Additional file 1: Fig. S1 The secondary structures and multiple sequence alignments of the two subunits of ScADAT2/3. [file 12915_2020_920_MOESM1_ESM.docx]

**
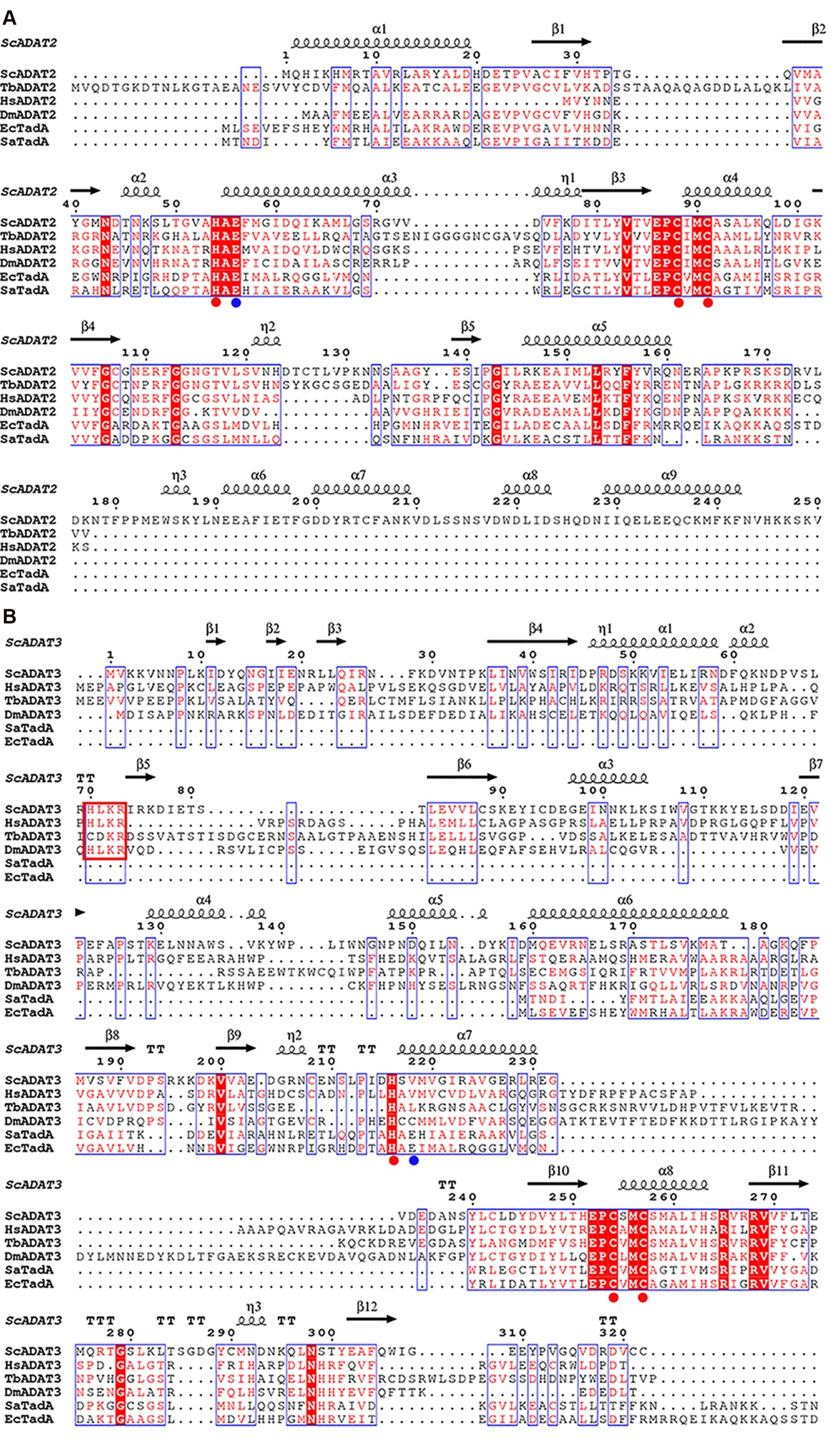
**

**
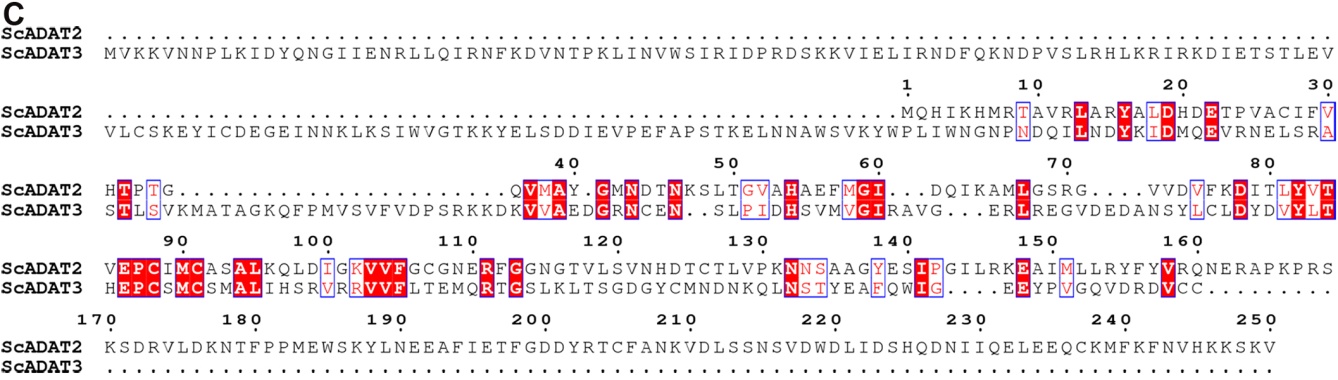
**

**Additional file 1: Fig. S1. The secondary structures and multiple sequence alignments of the two subunits of ScADAT2/3.** (**A)** ScADAT2 and homologs. (**B)** ScADAT3 and homologs. The ADATs from two bacterial species (EcTadA and SaTadA) were also included. The secondary structure elements were labeled above the sequences (based on chains B and D). The red dots indicated residues responsible for zinc binding, and the blue dots indicated the catalytic residues or the replacements. The positively charged tetra-peptide 70HLKR73 motif was indicated by the red box. Sc: *Saccharomyces cerevisiae*; Hs: *Homo sapiens*; Tb: *Trypanosoma brucei*; Dm: *Drosophila melanogaster*; Sp: *Schizosaccharomyces pombe*; Sa: *Staphylococcus aureus*; Ec: *Escherichia coli*. (**C**) Sequence alignments between ScADAT2 and ScADAT3.
