# Supplementary material for: Crystal structure of the yeast heterodimeric ADAT2/3 deaminase
Source: BMC Biol. 2020 Dec 3;18:189. doi: 10.1186/s12915-020-00920-2 (PMC7713142; doi:10.1186/s12915-020-00920-2)
Supplement: Supplementary file 2 — Additional file 2: Table S1 Data collection and refinement statistics. [file 12915_2020_920_MOESM2_ESM.docx]

**Additional file 2: Table S1. Data collection and refinement statistics.**

| Data collection | SSRF BL17U1 |
| --- | --- |
| Space group | *P*2_1_2_1_2 |
| a, b, c (Å) | 91.1, 118.5, 138.6 |
| α, β, γ (°) | 90, 90, 90 |
| Resolution (Å) | 50-2.8 (2.9-2.8)^a^ |
| R_merge_^b^ | 0.20 (0.82) |
| I/σ_(I)_ | 7.8 (1.4) |
| *CC*_1/2_ | 0.956 (0.574) |
| Completeness (%) | 99.7 (98.2) |
| Redundancy | 6.1 (4.0) |
| Refinement |  |
| Resolution (Å) | 50-2.8 (2.87-2.8) |
| No. reflections | 35555 |
| R_work_^c^/R_free_^d^ | 0.200/0.255 |
| No. atoms |  |
| Protein | 8097 |
| Ligand | 4 |
| Water | 108 |
| B-factors (Å ^2^) |  |
| Protein | 56.2 |
| Ligand | 35.4 |
| Water | 38.7 |
| R.m.s deviations |  |
| Bond lengths (Å) | 0.01 |
| Bond angles (º) | 1.56 |
| Ramachandran favored (%) | 94.0 |
| Allowed | 6.0 |
| Outliers (%) | 0.0 |

^a^Values in parentheses are for the highest-resolution shell. ^b^R_merge_ =Σ |(I - < I > )|/σ(I), where I is the observed intensity. ^c^R_work_ = Σ_hkl_ ||Fo| - |Fc||/ Σ_hkl_ |Fo|, calculated from working data set. ^d^R_free_ is calculated from 5.0% of data randomly chosen and not included in refinement.
