# Supplementary material for: Crystal structure of the yeast heterodimeric ADAT2/3 deaminase
Source: BMC Biol. 2020 Dec 3;18:189. doi: 10.1186/s12915-020-00920-2 (PMC7713142; doi:10.1186/s12915-020-00920-2)
Supplement: Supplementary file 3 — Additional file 3: Fig. S2 Purified WT ScADAT2/3 complexes and mutants used for crystallography, activity assays and EMSA. [file 12915_2020_920_MOESM3_ESM.docx]

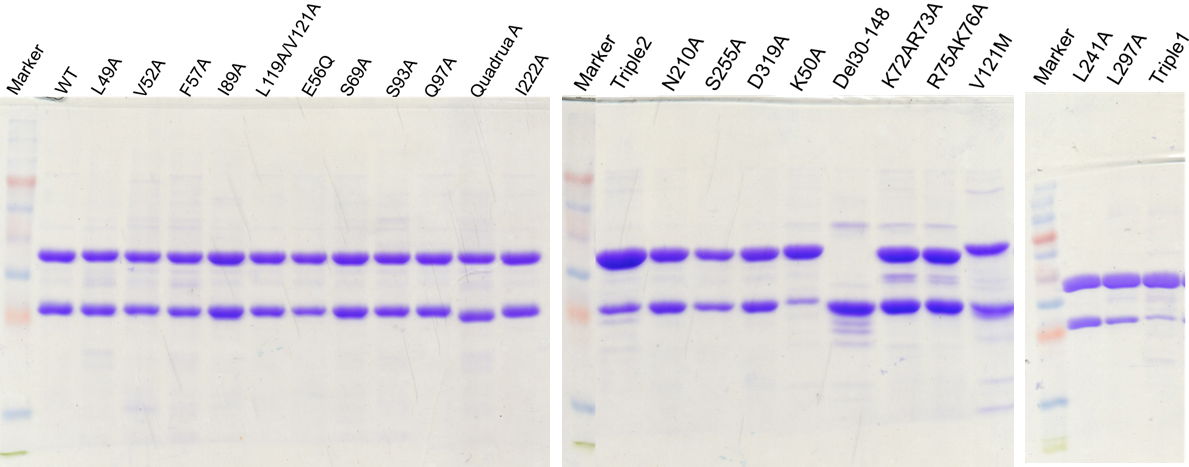


**Additional file 3: Fig. S2.** **Purified WT ScADAT2/3 complexes and mutants used for crystallography, activity assays and EMSA.**
