# Supplementary material for: Crystal structure of the yeast heterodimeric ADAT2/3 deaminase
Source: BMC Biol. 2020 Dec 3;18:189. doi: 10.1186/s12915-020-00920-2 (PMC7713142; doi:10.1186/s12915-020-00920-2)
Supplement: Supplementary file 4 — Additional file 4: Table S2 The quantification of the deamination activity assays. [file 12915_2020_920_MOESM4_ESM.docx]

**Additional file 4: Table S2. The quantification of the deamination activity assays.** The activities of the mutants were quantified as percentages of that of WT. Values were the means ± ranges of at least two separate experiments.

| Variants | Relative activity (%) |
| --- | --- |
| WT | 100 |
| ADAT2/E56Q | 1.3 ± 1.5 |
| ADAT2/S69A | 77.8 ± 2.5 |
| ADAT2/S93A | 85.0 ± 3.8 |
| ADAT2/Q97A | 61.3 ± 3.3 |
| ADAT2/QuadruA | 81.8 ± 4.1 |
| ADAT2/L49A | 98.9 ± 4.6 |
| ADAT2/V52A | 92.1 ± 4.5 |
| ADAT2/F57A | 57.1 ± 3.8 |
| ADAT2/I89A | 58.1 ± 3.4 |
| ADAT2/L119A/V121A | 76.4 ± 4.0 |
| ADAT3/V121M | 12.4 ± 2.3 |
| ADAT3/I222A | 93.65 ± 2.9 |
| ADAT3/L241A | 16.27 ± 1.4 |
| ADAT3/L297A | 20.20 ± 1.5 |
| ADAT3/TripleA1 | 14.11 ± 1.3 |
| ADAT3/TripleA2 | 16.12 ± 1.7 |
| ADAT3/N210A | 93.4 ± 4.3 |
| ADAT3/S255A | 90.0 ± 4.1 |
| ADAT3/D319A | 92.5 ± 5.0 |
| ADAT3/Del30-148 | 10.83% ±1.0 |
| ADAT3/K50A | 19.01 ±1.7 |
| ADAT3/ K72A/R73A | 91.05 ±3.1 |
| ADAT3/ R75A/K76A | 91.24 ±3.8 |
