# Supplementary material for: Crystal structure of the yeast heterodimeric ADAT2/3 deaminase
Source: BMC Biol. 2020 Dec 3;18:189. doi: 10.1186/s12915-020-00920-2 (PMC7713142; doi:10.1186/s12915-020-00920-2)
Supplement: Supplementary file 5 — Additional file 5: Fig. S3 schematic diagram showing the knockout/complementation protocols. [file 12915_2020_920_MOESM5_ESM.docx]

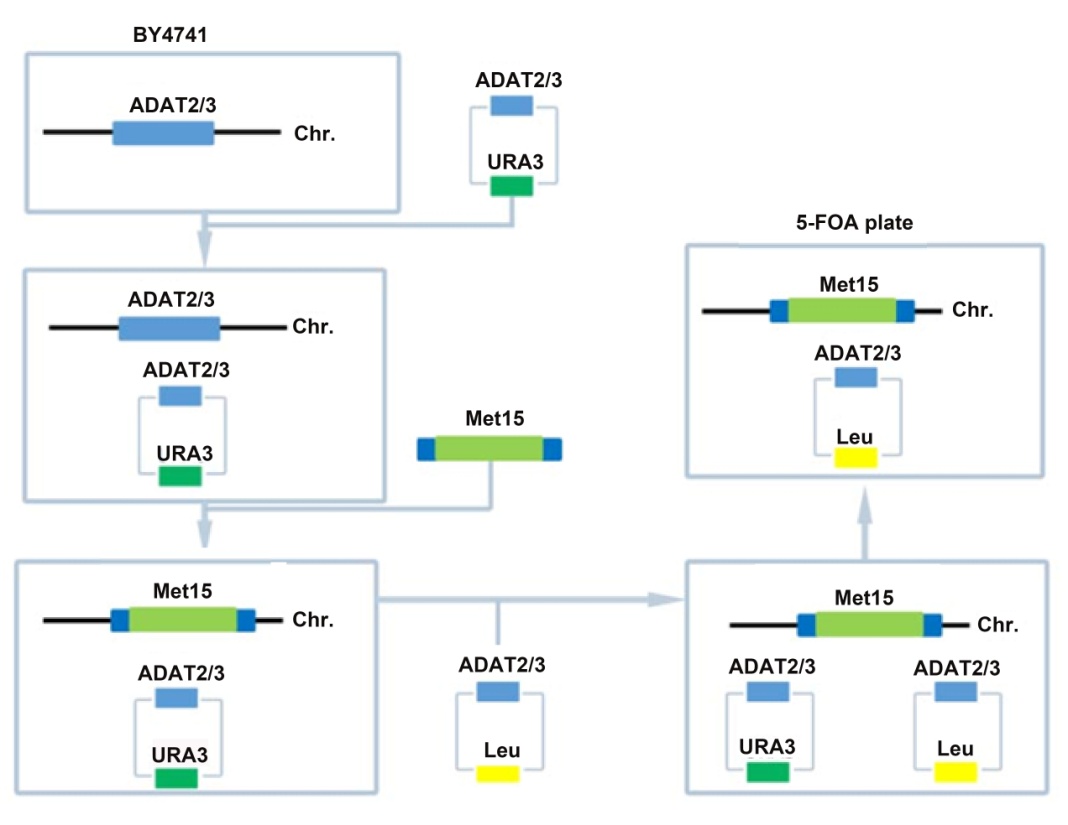


**Additional file 5: Fig. S3.** **A schematic diagram showing the knockout/complementation protocols.** The BY4741 strain was employed for the experiment.
