# Supplementary material for: Crystal structure of the yeast heterodimeric ADAT2/3 deaminase
Source: BMC Biol. 2020 Dec 3;18:189. doi: 10.1186/s12915-020-00920-2 (PMC7713142; doi:10.1186/s12915-020-00920-2)
Supplement: Supplementary file 6 — Additional file 6: Fig. S4 The circular dichroism spectra of WT ScADAT2/3 and mutants. [file 12915_2020_920_MOESM6_ESM.docx]

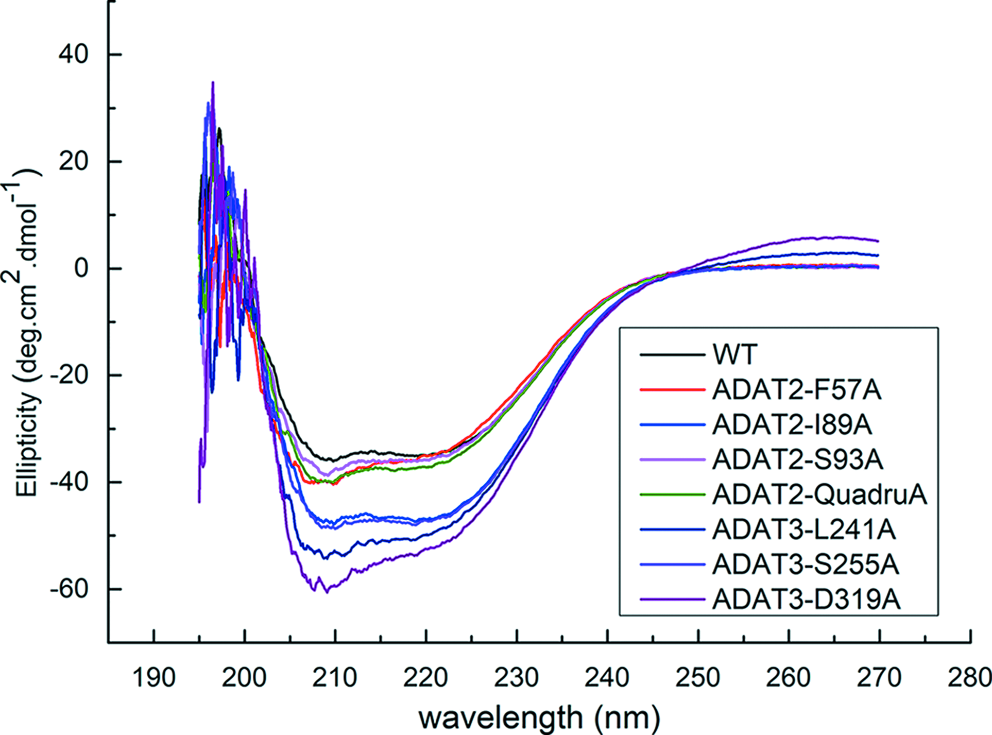


**Additional file 6: Fig. S4. The circular dichroism spectra of WT ScADAT2/3 and mutants.** The vertical axis represented ellipticity while the horizontal axis represented wavelength. The calculated fractions for each secondary structure elements were shown in Supplementary table 3.
