# Supplementary material for: Crystal structure of the yeast heterodimeric ADAT2/3 deaminase
Source: BMC Biol. 2020 Dec 3;18:189. doi: 10.1186/s12915-020-00920-2 (PMC7713142; doi:10.1186/s12915-020-00920-2)
Supplement: Supplementary file 7 — Additional file 7: Table S3 The circular dichroism analysis on the ScADAT2/3 mutants. [file 12915_2020_920_MOESM7_ESM.docx]

**Additional file 7: Table S3. The circular dichroism analysis on the ScADAT2/3 mutants.** Fractions for each secondary structure elements were calculated by CDpro.

| Secondary structure^a^ | H(r) | H(d) | S(r) | S(d) | Trn | Unrd |
| --- | --- | --- | --- | --- | --- | --- |
| WT | 0.265 | 0.209 | 0.116 | 0.141 | 0.137 | 0.136 |
| ADAT2/F57A | 0.264 | 0.215 | 0.109 | 0.144 | 0.135 | 0.142 |
| ADAT2/I89A | 0.209 | 0.186 | 0.140 | 0.152 | 0.151 | 0.165 |
| ADAT2/S93A | 0.224 | 0.197 | 0.138 | 0.150 | 0.143 | 0.152 |
| ADAT2/QuadruA | 0.251 | 0.209 | 0.113 | 0.139 | 0.135 | 0.165 |
| ADAT3/L241A | 0.225 | 0.195 | 0.130 | 0.152 | 0.149 | 0.152 |
| ADAT3/S255A | 0.224 | 0.194 | 0.137 | 0.154 | 0.146 | 0.154 |
| ADAT3/D319A | 0.190 | 0.179 | 0.152 | 0.161 | 0.159 | 0.170 |

^a^H(r): Regular helices (middle part); H(d): Distorted helices (the last two residues at each end); S(r): regular β-sheets (middle part); S(d): Distorted β-sheets (the last two residues at each end); Trn: β-turn; Unrd: others.
