# Supplementary material for: Crystal structure of the yeast heterodimeric ADAT2/3 deaminase
Source: BMC Biol. 2020 Dec 3;18:189. doi: 10.1186/s12915-020-00920-2 (PMC7713142; doi:10.1186/s12915-020-00920-2)
Supplement: Supplementary file 8 — Additional file 8: Fig. S5 The in-vitro tRNA deamination assay on potential tRNA-binding residues within RLD. [file 12915_2020_920_MOESM8_ESM.docx]

**
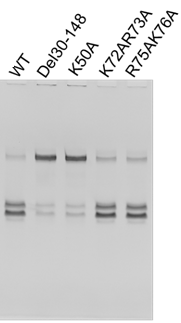
**

**Additional file 8: Fig. S5. The *in-vitro* tRNA deamination assay on potential tRNA-binding residues within RLD.** Del30-148: The deletion mutant with the removal of residues Asp30-Pro148 from ScADAT3.
