# Supplementary material for: Crystal structure of the yeast heterodimeric ADAT2/3 deaminase
Source: BMC Biol. 2020 Dec 3;18:189. doi: 10.1186/s12915-020-00920-2 (PMC7713142; doi:10.1186/s12915-020-00920-2)
Supplement: Supplementary file 9 — Additional file 9: Fig. S6 The co-expression/co-purification profile of the ScADAT2/3 WT and ScADAT2/3ΔCC truncation mutant. [file 12915_2020_920_MOESM9_ESM.docx]

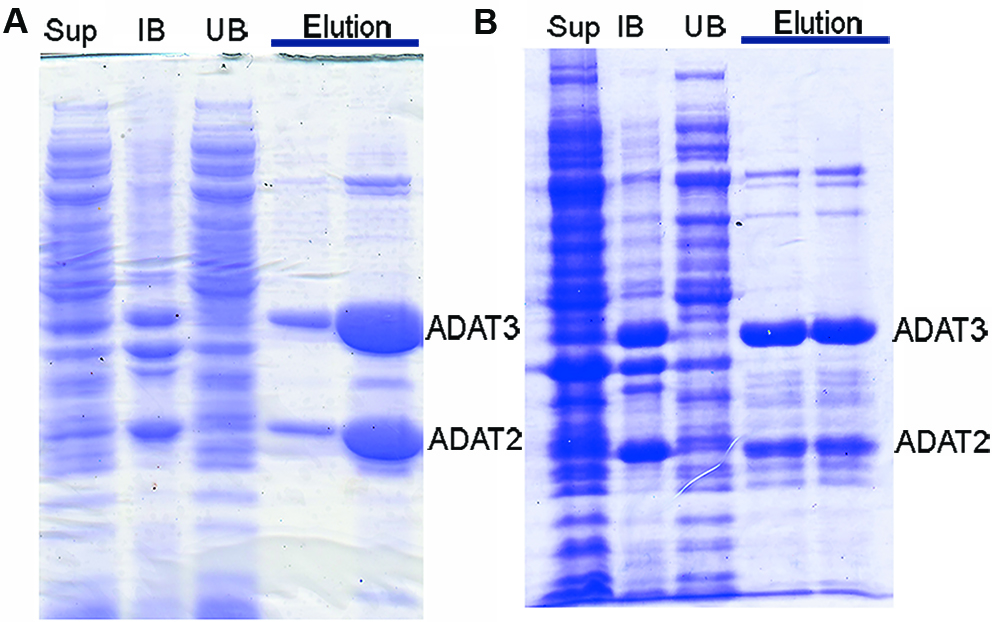


**Additional file 9: Fig. S6. The co-expression/co-purification profile of the ScADAT2/3 WT and ScADAT2/3ΔCC truncation mutant.** (**A**) ScADAT2/3WT. (**B**) ScADAT2/3ΔCC. The bands of the two subunits were indicated. Sup, the supernatant of ultrasonication after centrifugation; IB, the inclusion body after high-speed centrifugation; UB, the unbound fractions from Ni-NTA affinity column; Elution, the eluted fractions from the Ni-NTA column.
