# Supplementary material for: Crystal structure of the yeast heterodimeric ADAT2/3 deaminase
Source: BMC Biol. 2020 Dec 3;18:189. doi: 10.1186/s12915-020-00920-2 (PMC7713142; doi:10.1186/s12915-020-00920-2)
Supplement: Supplementary file 10 — Additional file 10: Table S4 Expression and ICP-MS profile of the Cys-to-Ser mutants of SpADAT3. [file 12915_2020_920_MOESM10_ESM.docx]

**Additional file 10: Table S4. Expression and ICP-MS profile of the Cys-to-Ser mutants of SpADAT3.**

|  | Expression profile | ICP-MS (Zinc/equivalent) |
| --- | --- | --- |
| WT | Normal expression | 1.0 |
| SpADAT3/C182S | Reduced expression | 1.0 |
| SpADAT3/C212S | Reduced expression | 1.4 |
| SpADAT3/C241S | No expression | NA* |
| SpADAT3/C256S | No expression | NA |
| SpADAT3/C271S | Reduced expression | 1.2 |

*: Not applicable.
