# Supplementary material for: Crystal structure of the yeast heterodimeric ADAT2/3 deaminase
Source: BMC Biol. 2020 Dec 3;18:189. doi: 10.1186/s12915-020-00920-2 (PMC7713142; doi:10.1186/s12915-020-00920-2)
Supplement: Supplementary file 12 — Additional file 12: Table S5 Sequences of the primers and tRNA used in this study. [file 12915_2020_920_MOESM12_ESM.docx]

**Additional file 12: Table S5. Sequences of the primers and tRNA used in this study.** The restriction sites of the cloning primers were underlined in the amplifying primers while the mutated bases were in italic in the *Quikchange* primers.

| Clones or mutations | Sequence (5’-3’) |
| --- | --- |
| ADAT3-F1 | GCTTAGGAATTCGATGGTTAAGAAAGTT |
| ADAT3-R1 | ATAGGAAAGCTTTCAGCAGCAGACATCCCG |
| ADAT2-F1 | CATATGGCAGATCTCCAGCATATTAAACAT |
| ADAT2-R1 | TTGCACTTCTCGAGTCAAACCTTAGATTTCCTTATG |
| ADAT3-F2 | ATAGGAGCTAGCATGGTTAAGAAAGTTAATAAT |
| F-ADAT2-L49A | ATACGAACAAGTCA*GC*AACTGGAGTAGCAC |
| R-ADAT2-L49A | GTGCTACTCCAGTTGCTGACTTGTTCGTAT |
| F-ADAT2-V52A | TCATTAACTGGAG*C*AGCACATGCTGAG |
| R-ADAT2-V52A | CTCAGCATGTGCTGCTCCAGTTAATGA |
| F-ADAT2-F57A | TAGCACATGCTGAG*GC*CATGGGGATCGATC |
| R-ADAT2-F57A | GATCGATCCCCATGGCCTCAGCATGTGCTA |
| F-ADAT2-S69A | GCGATGTTGGGC*G*CCCGAGGAGTTG |
| R-ADAT2-S69A | CAACTCCTCGGGCGCCCAACATCGC |
| F-ADAT2-I89A | CTGTAGAACCGTGT*GC*AATGTGTGCATCTG |
| R-ADAT2-I89A | CAGATGCACACATTGCACACGGTTCTACAG |
| F-ADAT2-S93A | ATAATGTGTGCA*G*CTGCTCTCAAGC |
| R-ADAT2-S93A | GCTTGAGAGCTGCTGCACACATTAT |
| F-ADAT2-Q97A | CATCTGCTCTCAAG*GC*ATTAGACATTGGAA |
| R-ADAT2-Q97A | TTCCAATGTCTAATGCCTTGAGAGCAGATG |
| F-ADAT2-L119A/V121A | GTCA*GC*AAATCATGATACGTGTACA |
| R-ADAT2-L119A/V121A | *GC*GACAGTACCGTTGCCTCC |
| F-ADAT2-D124A/T125A/C126A/T127A (QuadruA) | G*CAG*CG*GCAG*CATTAGTGCCCAAGAACAAT |
| R-ADAT2- D124A/T125A/C126A/T127A  (QuadruA) | ATGATTTACTGACAAGACAGT |
| F-ADAT3- V121M | TGATGATATCGAA*A*TGCCTGAGTTTGC |
| R-ADAT3- V121M | GCAAACTCAGGCATTTCGATATCATCA |
| F-ADAT3-N210A | CAGAAACTGCGAA*GC*CTCGCTACCCATC |
| R-ADAT3-N210A | GATGGGTAGCGAGGCTTCGCAGTTTCTG |
| F-ADAT3-I222A | TGTAATGGTGGGC*GC*CCGTGCGGTAGGC |
| R-ADAT3-I222A | GCCTACCGCACGGGCGCCCACCATTACA |
| F-ADAT3-L241A | CGCAAACTCTTAT*GC*GTGTCTTGATTAC |
| R-ADAT3-L241A | GTAATCAAGACACGCATAAGAGTTTGCG |
| F-ADAT3-S255A | CATGAGCCGTGC*G*CAATGTGCTCCA |
| R-ADAT3-S255A | TGGAGCACATTGCGCACGGCTCATG |
| F-ADAT3-M259A | GCTCAATGTGCTCC*GC*GGCCCTGATCCATT |
| R-ADAT3-M259A | AATGGATCAGGGCCGCGGAGCACATTGAGC |
| F-ADAT3-L297A | CGATAACAAACAG*GC*AAACTCAACATAC |
| R-ADAT3-L297A | GTATGTTGAGTTTGCCTGTTTGTTATCG |
| F-ADAT3-D319A | AGGTTGACCGGG*C*TGTCTGCTGCTC |
| R-ADAT3-D319A | GAGCAGCAGACAGCCCGGTCAACCT |
| F-ADAT3-L212A/P213A/I214A (TripleA1) | *GC*A*G*CC*GC*CGACCACAGTGTAATGGTG |
| R-ADAT3-L212A/P213A/I214A (TripleA1) | CGAGTTTTCGCAGTTTCTG |
| F-ADAT3-D287A/Y289A/M291A (TripleA2) | *C*CGGG*GC*CTGC*GC*GAACGATAACAAACAGCTAAAC |
| R-ADAT3-D287A/Y289A/M291A (TripleA2) | CACCACTCGTTAATTTTAAGC |
| F-ADAT3-△CC | TGAAAGCTTGCGGCCGCATAA |
| R-ADAT3-△CC | GACATCCCGGTCAACCTGCC |
| F-Mel15-ADAT3 | GTATGTATCTATGAGATCTAACAGAAATCAGGATCAATTAACTTATTTTTTGCTTTTTCT |
| R-Mel15-ADAT3 | CAGTTCCTTTGTGCTGGGCGCAAACTCAGGCACTTCGATATC TCATGGTTTTTGGCCAGC |
| F-Mel15-ADAT2 | TACATTAGTGCCCAAGAACAATAGTGCGGCAGGGTACGAGAGTTATTTTTTGCTTTTTCT |
| R-Mel15-ADAT 2 | TGTAAATGTTGCTATTATTTTATGATGTTCTGATTTTTTTCG TCATGGTTTTTGGCCAGC |
| F-ADAT2-ide | TGTCCAGTTCCTCGGGTGCC |
| R-ADAT2-ide | GTGCTCTTAATGTTTTCCCA |
| F-ADAT3-ide | ATGGTTAAGAAAGTTAATAA |
| R-ADAT3-ide | GCCTACCGCACGGATGCCCACCATTACA |
| tRNA^Ala^ | GGGCGUGUGGCGUAGUCGGUAGCGCGCUCCCUUAGCAUGGGAGAGGUCUCCGGUUCGAUUCCGGACUCGUCCACCA |
| F-SpADAT3-C182S | GGCGAAATAGGAT*C*TGCTGCTGCTATT |
| R-SpADAT3-C182S | AATAGCAGCAGCAGATCCTATTTCGCC |
| F-SpADAT3-C212S | CCTATAAACCACTCTGTGATGAATGCC |
| R-SpADAT3-C212S | GGCATTCATCACA*G*AGTGGTTTATAGG |
| F-SpADAT3- C241S | GACTCATGAACCAT*CG*GTAATGTGTAGTAT |
| R-SpADAT3- C241S | ATACTACACATTACCGATGGTTCATGAGTC |
| F-SpADAT3-C256S | ACCATGTGTAATG*A*GTAGTATGGGTCT |
| R-SpADAT3-C256S | AGACCCATACTACTCATTACACATGGT |
| F-SpADAT3-C271S | GCGTTTAATTTAC*A*GCAAAAAGCAACC |
| R-SpADAT3-C271S | GGTTGCTTTTTGCTGTAAATTAAACGC |
